# Characterization of erythroferrone structural domains relevant to its iron-regulatory function

**DOI:** 10.1016/j.jbc.2023.105374

**Published:** 2023-10-20

**Authors:** Daniel N. Srole, Grace Jung, Alan J. Waring, Elizabeta Nemeth, Tomas Ganz

**Affiliations:** 1 Department of Medicine, Center for Iron Disorders, David Geffen School of Medicine at UCLA, Los Angeles, California, USA; 2 Molecular and Medical Pharmacology Graduate Program, Graduate Programs in Bioscience, Los Angeles, California, USA; 3 Department of Medicine, Harbor-UCLA Medical Center, Lundquist Institute, Los Angeles, California, USA

**Keywords:** iron, erythropoiesis, bone morphogenetic protein (BMP), hepcidin, erythroferrone, surface plasmon resonance (SPR), molecular modeling

## Abstract

Iron delivery to the plasma is closely coupled to erythropoiesis, the production of red blood cells, as this process consumes most of the circulating plasma iron. In response to hemorrhage and other erythropoietic stresses, increased erythropoietin stimulates the production of the hormone erythroferrone (ERFE) by erythrocyte precursors (erythroblasts) developing in erythropoietic tissues. ERFE acts on the liver to inhibit bone morphogenetic protein (BMP) signaling and thereby decrease hepcidin production. Decreased circulating hepcidin concentrations then allow the release of iron from stores and increase iron absorption from the diet. Guided by evolutionary analysis and Alphafold2 protein complex modeling, we used targeted ERFE mutations, deletions, and synthetic ERFE segments together with cell-based bioassays and surface plasmon resonance to probe the structural features required for bioactivity and BMP binding. We define the ERFE active domain and multiple structural features that act together to entrap BMP ligands. In particular, the hydrophobic helical segment 81 to 86 and specifically the highly conserved tryptophan W82 in the N-terminal region are essential for ERFE bioactivity and Alphafold2 modeling places W82 between two tryptophans in its ligands BMP2, BMP6, and the BMP2/6 heterodimer, an interaction similar to those that bind BMPs to their cognate receptors. Finally, we identify the cationic region 96-107 and the globular TNFα-like domain 186-354 as structural determinants of ERFE multimerization that increase the avidity of ERFE for BMP ligands. Collectively, our results provide further insight into the ERFE-mediated inhibition of BMP signaling in response to erythropoietic stress.

Iron is an essential micronutrient required for many cellular and organismal processes in nearly all living organisms. Experimental studies in humans and laboratory rodents have shown that the absorption, storage, and transport of iron atoms are tightly regulated. Hepcidin—a small peptide hormone produced by the liver—controls the movement of iron into plasma by occluding and internalizing its receptor, the cellular iron exporter ferroportin (1). Numerous stimuli regulate the transcription of the hepcidin gene *HAMP*, either to induce or to suppress hepcidin production. Iron loading in the liver increases hepcidin secretion by hepatocytes by stimulating sinusoidal endothelial cells to produce bone morphogenetic proteins (BMPs) which act on hepatocytes to induce *HAMP* transcription *via* the BMP–SMAD pathway (2). Inflammation induces *HAMP* transcription *via* interleukin 6 signaling through the Janus kinase-signal transducer and activator of transcription pathway (3, 4). Hypoxia is sensed in the kidneys in specialized erythropoietin (EPO)-producing cells to increase hypoxia inducible factor-2α and thereby stimulate EPO production. EPO then acts on the marrow to stimulate erythropoiesis and to increase the secretion of erythroid factors that act on the liver to lower *HAMP* transcription, decrease hepcidin protein levels, and make iron available for erythropoiesis (5).

Erythroferrone (ERFE) is produced by erythroblasts in the marrow or spleen in response to EPO signaling. By inhibition of the hepatic BMP–SMAD signaling axis, ERFE suppresses hepcidin transcription in the liver to decrease circulating hepcidin concentrations and thereby increase iron absorption from the diet and to mobilize iron from stores in hepatocytes and macrophages. Apart from the physiological role of ERFE in erythropoietic recovery, ERFE also plays a pathological role in anemias with ineffective erythropoiesis (*e.g.* β-thalassemia) where excessive production of ERFE, as a result of high EPO levels and a greatly expanded population of erythroblasts, contributes to the development of iron overload (6, 7).

Regarding the mechanism of action of ERFE, surface plasmon resonance (SPR) and competition studies have demonstrated that ERFE binds to and neutralizes a subset of BMP ligands including dimeric BMP2, BMP6, and BMP2/6 (8, 9) and that the N terminus of ERFE is sufficient for its bioactivity. The posttranslational modifications of ERFE that affect its folding, secretion, and multimerization have also been analyzed (10) but the effects of these structural features on ERFE bioactivity have not been examined.

In the current study, we performed molecular characterization of the human ERFE protein to identify features that are important for its hepcidin-regulating activity and therefore its (patho)physiological role in iron mobilization. Using protein modeling and docking models, mutagenesis, cell-based bioassays, and SPR, we identified the ERFE active domain, structural features, and amino acid residues that are key to its function and generated an updated model of the ERFE structure-function relationship.

## Results

### Structure-function study of the N and C terminus of ERFE

**Figure 1:**
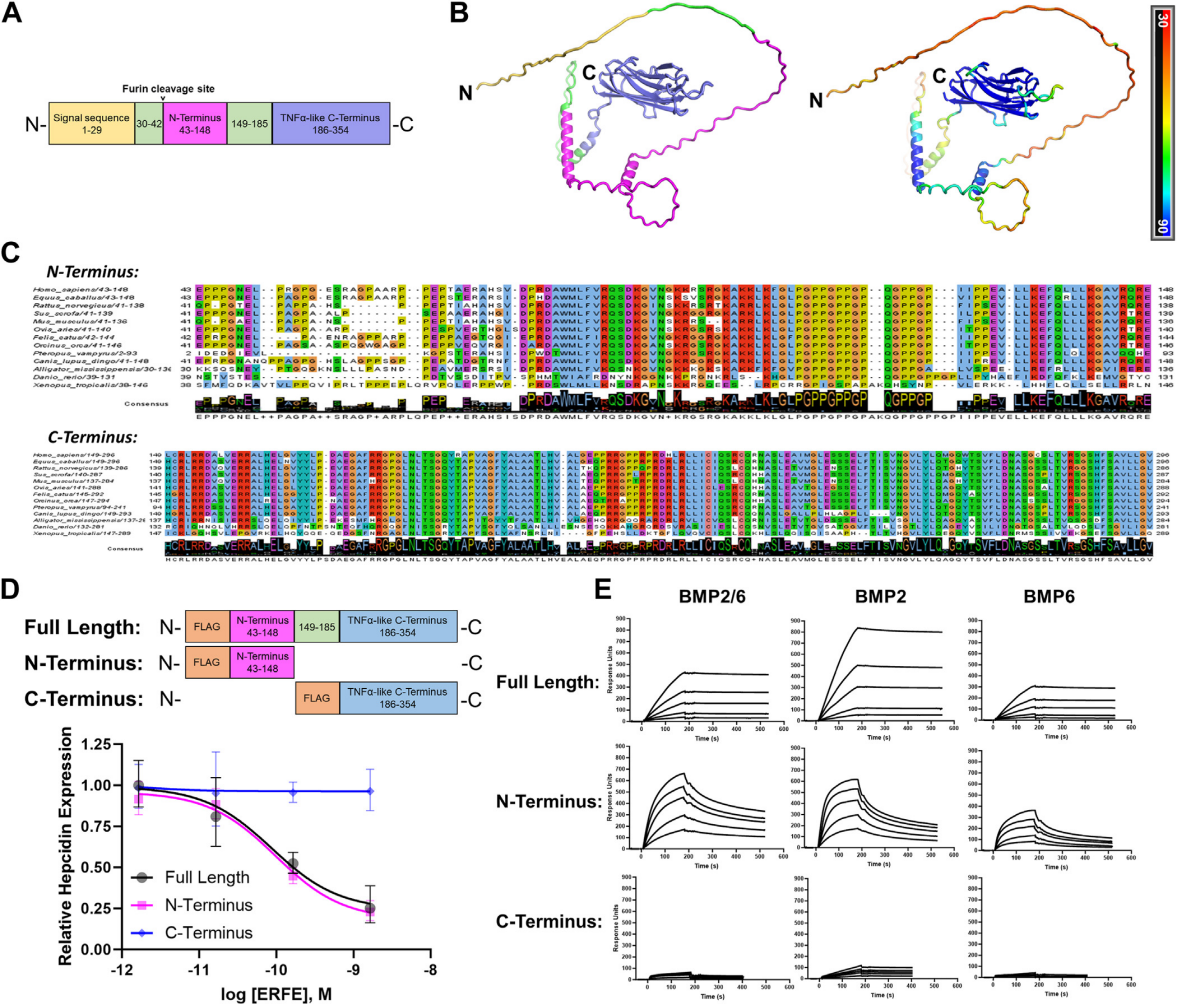
**Structure-function study of the N and C terminus of ERFE.***A*, diagram of the human ERFE protein showing the signal sequence, N-terminal, and C-terminal domains, and their respective amino acid boundaries. *B*, AlphaFold2 model of human ERFE structure (*left*; colors correspond to segments in the diagram A and *right*; colored by confidence parameter pLDDT, *blue* = highest degree of confidence). *C*, amino acid conservation alignments of ERFE N-terminal and C-terminal domains across vertebrate species. *D*, *top*: diagrams of full-length, N terminus, and C terminus ERFE constructs that were used in bioassay and SPR. *Bottom*: qPCR expression of hepcidin in Hep3B cells treated with a range of concentrations of ERFE variants for 16 h. Data are normalized to untreated controls. N = 3 biological replicates. *E*, surface plasmon resonance sensorgrams of full-length, N-terminal, and C-terminal ERFE binding to immobilized BMP2, BMP6, and BMP2/6. BMP, bone morphogenetic protein; ERFE, erythroferrone; pLDDT, predicted local distance difference test; SPR, surface plasmon resonance.

Like all members of the C1q/TNFα-related protein family (CTRP), ERFE is predicted to contain a leading signal sequence that directs it for secretion, a variable N-terminal region, and a TNFα-like head at the C terminus (Fig. 1*A*). We used AlphaFold2 to generate a model of the human ERFE structure. As expected, the algorithm predicts a highly structured C terminus and mostly unstructured N terminus (Fig. 1*B*). Within the N terminus, the model contains three small alpha helices but no other well-defined structural elements. These helices along with the C-terminal head are predicted with the highest confidence, while most of the N terminus is generated with low or very low confidence suggesting that this portion of the protein is disordered.

The mature human N terminus is predicted to begin at Glu43 following a RARR PCSK3/furin recognition site which results in cleavage of the upstream region from the mature protein. The alignment of vertebrate ERFE sequences reveals that large parts of the N terminus and nearly the entire C terminus are remarkably conserved (Fig. 1*C*). Despite the disordered nature of the N terminus, several distinct structural features within it—distinguished by charge or polarity—appear to be retained across the range of vertebrate species.

To explore the functional properties of the ERFE domains, we designed constructs representing the N-terminal portion (amino acids 43–148), C-terminal portion corresponding to the globular head (186–354), and a full-length construct that represents the mature molecule (43–354). All three constructs use a pcDNA3.1 backbone and contain the ERFE signal sequence that is cleaved during secretion, followed by a retained FLAG tag upstream of the coding sequence (Fig. 1*D*, top).

To assess bioactivity of the ERFE fragments, we overexpressed full-length, N-terminal, and C-terminal ERFE segments using HEK293T cells (Fig. S1), fibroblast-like cells known to produce many human proteins with their posttranslational modifications, and determined the molar concentration of the proteins by quantitative Western blotting. We treated the human hepatocyte Hep3B cell line, which models many functions of primary human hepatocytes, with serial dilutions of ERFE-containing supernatants (Fig. 1*D*, bottom). Like full-length ERFE, the N-terminal segment potently suppressed hepcidin transcription, in agreement with published studies (8). The C-terminal segment alone did not exhibit bioactivity in this assay.

ERFE has previously been shown to interact with BMPs (8, 9, 11), and sequestration of BMPs from the BMP receptors may be the mechanism of action of hepcidin suppression. We used SPR to determine binding avidities for BMP species that are known to be important for hepcidin induction (12, 13): a heterodimer of BMP2/6 and homodimers of BMP2 and BMP6 (Fig. 1*E*). We use the term “avidity” for functional affinity when the protein–protein interactions are presumed multivalent and “affinity” when they are expected to be univalent. Mirroring hepcidin suppression, the ERFE N terminus and the full-length protein bind to these BMPs, whereas the C terminus does not bind. Our data confirm that the relatively unstructured N-terminal segment but not the highly structured C-terminal portion of ERFE is required for both binding to BMPs and for hepcidin suppression. Nevertheless, while the C terminus does not interact with any BMP tested, its presence in the full-length protein increases avidity for the ligands as demonstrated by more than an order-of-magnitude higher avidity for BMPs than the N terminus alone (Table 1).

**Table 1:** ERFE-BMP equilibrium dissociation constant (K_d_ (M)) and pK_d_ (pK_d_ = -log K_d_, mean ± SD, n = 5)

ERFE variant	BMP2/6	BMP2	BMP6
Full length, 43-354	2.77 × 10^−10^	4.45 × 10^−10^	8.17 × 10^−10^
	9.6 ± 0.3	9.5 ± 0.4	9.5 ± 0.6
N terminus (mammalian), 43-148	5.38 × 10^−9^	7.84 × 10^−9^	5.46 × 10^−9^
	8.3 ± 0.2	8.1 ± 0.2	8.3 ± 0.3
N terminus (bacterial), 43-148	5.8 × 10^−9^	3.41 × 10^−8^	2.49 × 10^−8^
	8.3 ± 0.2	7.5 ± 0.1	8.8 ± 0.4
W82A, 43-148	1.89 × 10^−8^	8.99 × 10^−8^	1.84 × 10^−7^
	7.7 ± 0.1	7.1 ± 0.2	6.7 ± 0.1
6KA mutant, 43-148	4.27 × 10^−9^	2.42 × 10^-7^	6.56 × 10^−9^
	8.37 ± 0.02	6.62 ± 0.04	8.2 ± 0.1
KR switch, 43-148	2.57 × 10^−9^	5.04 × 10^-8^	1.84 × 10^−9^
	8.6 ± 0.1	7.3 ± 0.2	8.8 ± 0.2
ΔCollagen, 43-148 del109-125	5.01 × 10^−8^	3.13 × 10^−9^	7.56 × 10^−10^
	7.30 ± 0.03	8.7 ± 0.5	9.2 ± 0.2
Δhelical, 43-125	8.52 × 10^−8^	7.50 × 10^−8^	1.16 × 10^−7^
	7.1 ± 0.3	7.1 ± 0.2	7.0 ± 0.1
Segment 1, 43-95	no binding	no binding	no binding
Segment 2, 73-125	6.61 × 10^−6^	1.96 × 10^−6^	1.67 × 10^−7^
	5.2 ± 0.3	5.8 ± 0.4	6.78 ± 0.01
Segment 3, 96-148	7.75 × 10^−6^	1.48 × 10^−5^	5.03 × 10^−7^
	5.4 ± 0.6	4.9 ± 0.3	6.4 ± 0.3

### The N-terminal active domain contains multiple BMP-binding features

**Figure 2:**
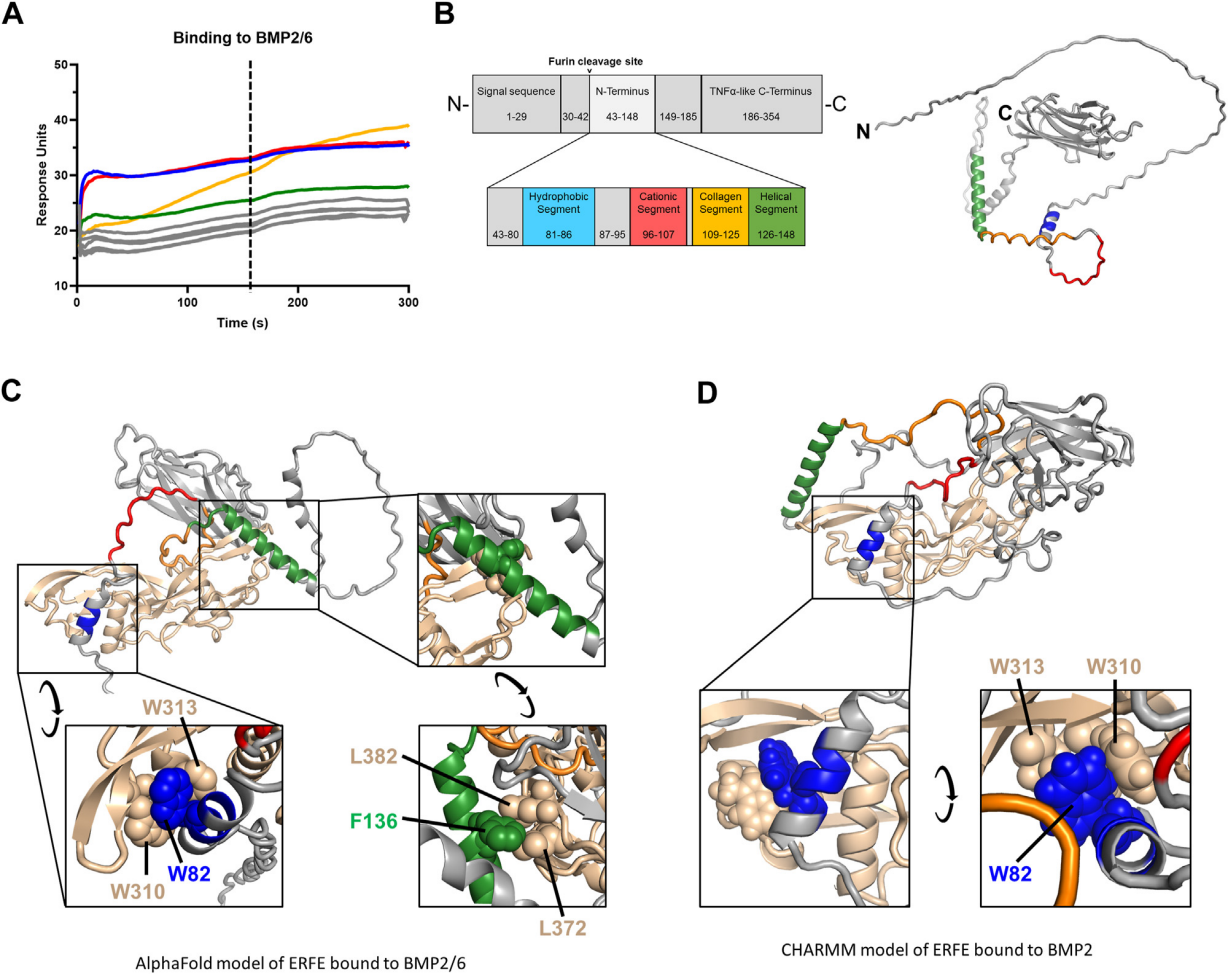
**The ERFE N terminus is comprised of multiple functional segments.***A*, synthetic 18-mer peptides that span the ERFE N terminus were tested by surface plasmon resonance for binding to BMP2/6, with a 180 s injection phase (end indicated by *vertical* dashed line) and subsequent buffer wash. Peptide sequences and color-coding are shown in Fig. S2*A*. The color corresponds to the specific ERFE structural segments shown in *B*. *B*, diagram of the ERFE N-terminal structural segments (*left*) and AlphaFold2 model of ERFE with N-terminal segments indicated by corresponding color (*right*). *C*, AlphaFold2 model of ERFE bound to a BMP2/6 heterodimer colored by segment. Callout boxes show potential interactions using aromatic residues of the ERFE hydrophobic and helical segments. *D*, molecular dynamics–refined model of ERFE bound to a BMP2 homodimer. Callout box shows potential interactions involving aromatic residues of the ERFE hydrophobic segment. BMP, bone morphogenetic protein; ERFE, erythroferrone.

We next generated overlapping 18-mer peptides that span the N-terminal domain of human ERFE (Fig. S2*A*) and used SPR to detect their binding to BMP2/6, BMP2, or BMP6 (Fig. 2*A* and S2*B*). Four of the peptides (73–90, 97–114, 121–138, and 133–150) interacted with BMP ligands, indicating that ERFE may contain multiple potential contact points for BMPs. Based on these four peptides and distinct features of the ERFE protein sequence, we focused on four regions in the N terminus: the hydrophobic segment (81–86), the cationic segment (96–107), the collagen segment (109–125), and the helical segment (126–148) (Fig. 2*B*).

To model how these segments could interact with BMPs, we used AlphaFold2 to dock the full-length ERFE protein to a BMP2/6 dimer (Fig. 2*C*, see also Data S1 for model quality measures). BMP2 and BMP6 homodimers interacted with ERFE in a similar manner except for the position of the globular head which was highly variable (Fig. S2, *D* and *E*). In all three situations, two of the four segments were predicted by the model to directly interact with the BMP: the hydrophobic segment containing a conserved tryptophan (W82) that appears to interact with two tryptophans of BMP2 (W310 and W313) and the helical segment which has a conserved phenylalanine (F136) interacting with two BMP leucines (L372 and L382). In this docking model, the predicted local distance difference test scores of the local structure are higher at these interaction sites than anywhere else in the N terminus but the interaction at the helical segment appears much more variable (Fig. S2, *C*–*E*).

To validate our findings by an independent method, we generated a second model of the same ERFE–BMP2 dimer interaction using a molecular dynamics–refined coordinate set (13).

This second model predicts an identical interaction in the hydrophobic segment between the same residues of ERFE and BMP2 as AlphaFold2 (Fig. 2*D*). A second contact between the ERFE helical segment and BMP wing is present in this model, but it does not exactly match the residues identified by AlphaFold2. Neither model predicts the interactions of the cationic or collagen segments with BMP that were detected by our SPR 18-mer peptide scan. The discrepancy is not surprising as the 18-mer peptides are small and flexible so could make more contacts with BMPs than the 3-dimensional folded full-length protein. The AlphaFold2 and molecular dynamics–refined models also differ in their positioning of the C terminus, but both agree with our experimental finding that the region has no direct interaction with BMPs.

### The conserved tryptophan in the hydrophobic segment is essential for activity

**Figure 3:**
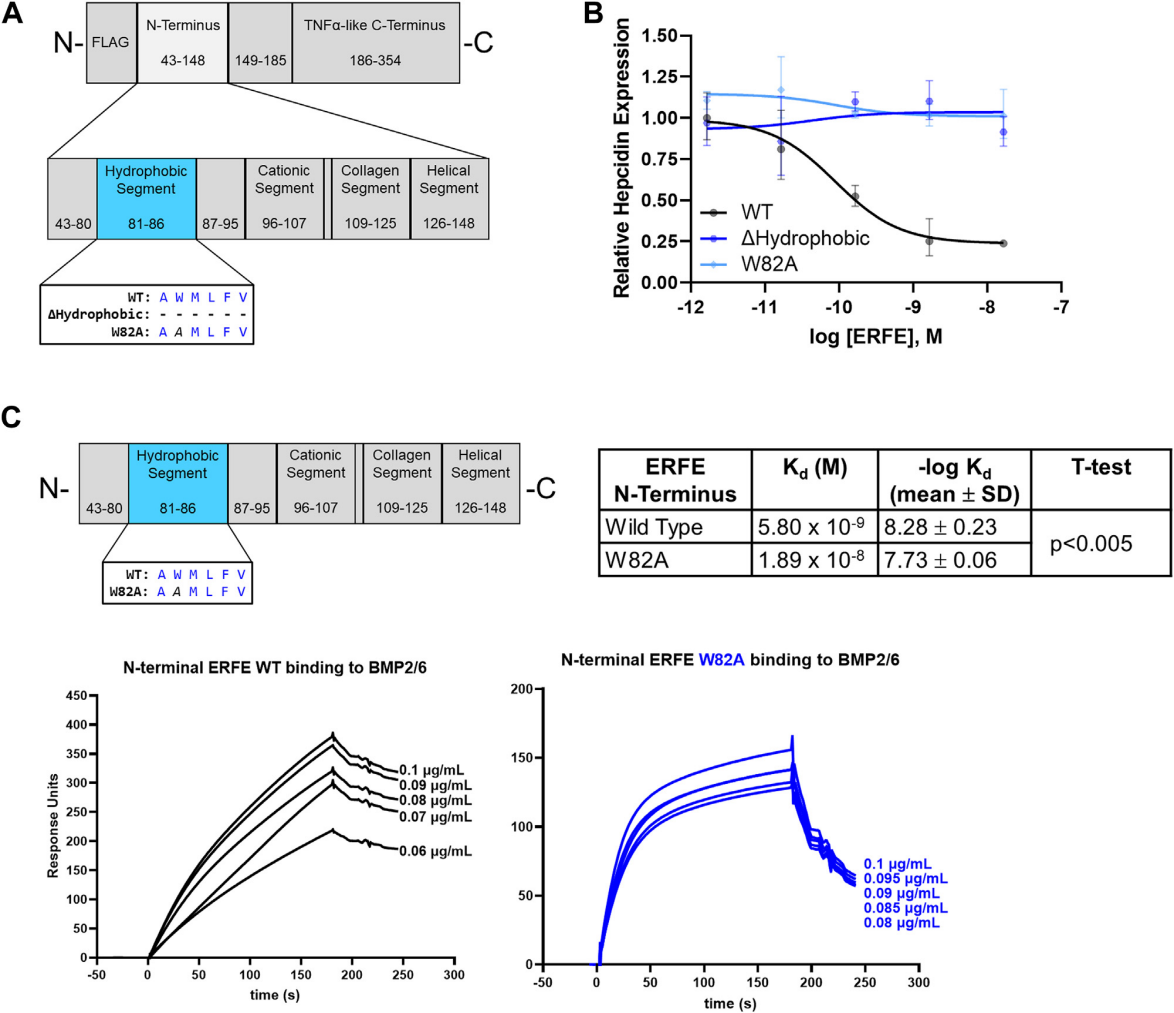
**The hydrophobic segment of ERFE N terminus.***A*, diagram of two mutations in the ERFE hydrophobic segment used for bioassay: deletion of amino acids 81 to 86 (ΔHydrophobic) and a single amino acid substitution W82A. *B*, qPCR expression of hepcidin in Hep3B cells treated with indicated concentrations of WT, ΔHydrophobic, and W82A ERFE. Data are normalized to untreated controls, N = 3 biological replicates, statistics in Table 3. *C*, surface plasmon resonance sensorgrams of ERFE WT and W82A N-terminus binding to BMP2/6, with mean K_d_ and -log K_d_ of each shown on the *right*, compared by *t* test to WT (n = 5). BMP, bone morphogenetic protein; ERFE, erythroferrone.

The ERFE vertebrate protein sequence alignment revealed that while a segment very similar to the human ERFE region 81-86 is universally present, only the tryptophan at position 82 (human numbering) is invariant (Fig. 1*C*). To test the function of this segment, we deleted the entire conserved segment (ΔHydrophobic) or introduced a point mutation of this tryptophan to alanine (W82A) to the full-length protein produced in HEK293T cells (Fig. 3*A*). Both mutants completely lost activity in the hepcidin-suppression assay (Fig. 3*B*), indicating a critical role of the tryptophan residue and the hydrophobic segment in ERFE’s mechanism of action. Since the tryptophan is predicted by both AlphaFold2 and molecular dynamics–refined coordinate set to interact directly with BMPs, we tested how well the W82A mutant binds BMPs. To avoid the confounding effects of ERFE multimerization driven by the TNFα-like globular regions on avidity, we purified bacterially produced, untagged ERFE N-terminal (43–148) segments—WT and W82A—and measured their binding to BMP2/6, 2, or 6 by SPR (Figs. 3*C* and S3, *A* and *B*). ERFE has multiple glycosylation sites that would not be correctly modified in bacteria; however, these sites are not part of the N terminus (10) which is tested by SPR. The W82A mutant bound BMPs(Figs. 3*C* and S3, *A* and *B*); however, it showed about 3-fold lower affinity (for BMP2/6 equilibrium, dissociation constant K_d_ 1.9 × 10^−8^ ± 2.7 × 10^−9^ *versus* 5.8 × 10^−9^ ± 2.8 × 10^−9^, *p* = 0.00007 by *t* test) and considerably faster dissociation rates (k_d_) than WT (k_d_ 0.029 ± 0.0008/s compared to 0.0051 ± 0.0002/s, mean ± SD, n = 5, *p* = 2 × 10^−6^ by 2-tailed unpaired *t* test), see also Tables 1 and 2). This implies that strong and persistent binding to BMPs favors the hepcidin-suppressive activity of ERFE.

**Table 2:** ERFE-BMP dissociation rates (k_d_ (1/s) and log k, mean ± SD, n = 5)

ERFE variant	BMP2/6	BMP2	BMP6
Full length, 43-354	0.00022 ± 0.00014	0.00009 ± 0.00002	0.00022 ± 0.00018
	−3.73 ± 0.30	−4.05 ± 0.11	−3.79 ± 0.41
N terminus (mammalian), 43-148	0.0022 ± 0.0003	0.0050 ± 0.0003	0.0042 ± 0.0002
	−2.67 ± 0.05	−2.30 ± 0.02	−2.38 ± 0.02
N terminus (bacterial), 43-148	0.0051 ± 0.0002	0.0046 ± 0.0002	0.0052 + 0.0003
	−2.29 ± 0.02	−2.34 ± 0.02	−2.29 ± 0.03
W82A, 43-148	0.024 ± 0.001	0.016 ± 0.008	0.029 ± 0.008
	−1.63 ± 0.02	−1.84 ± 0.24	−1.56 ± 0.12
6KA mutant, 43-148	0.029 ± 0.001	0.026 ± 0.002	0.029 ± 0.002
	−1.54 ± 0.01	−1.59 ± 0.004	−1.53 ± 0.02
KR switch, 43-148	0.0068 ± 0.0002	0.0058 ± 0.0008	0.0069 + 0.0005
	−2.17 ± 0.01	−2.24 ± 0.06	−2.16 ± 0.03
ΔCollagen, 43-148 del109-125	0.0052 ± 0.0002	0.0056± 0.0031	0.0085 ± 0.0076
	−2.29 ± 0.02	−2.29 ± 0.20	−2.17 ± 0.30
ΔHelical, 43-125	0.033 ± 0.001	0.034± 0.002	0.045 + 0.002
	−1.48 ± 0.01	−1.47 ± 0.02	−1.35 ± 0.02
Segment 1, 43-95	no binding	no binding	no binding
Segment 2, 73-125	0.016 ±0.003	0.018 ± 0.004	0.027 + 0.005
	−1.79 ± 0.07	−1.76 ± 0.09	−1.57 ± 0.08
Segment 3, 96-148	0.019 ± 0.005	0.027 ± 0.004	0.032 + 0.005
	−1.74 ± 0.12	−1.57 ± 0.07	−1.50 ± 0.06

### Clustered positive charge in the cationic segment mediates biological activity and interaction with heparin

**Figure 4:**
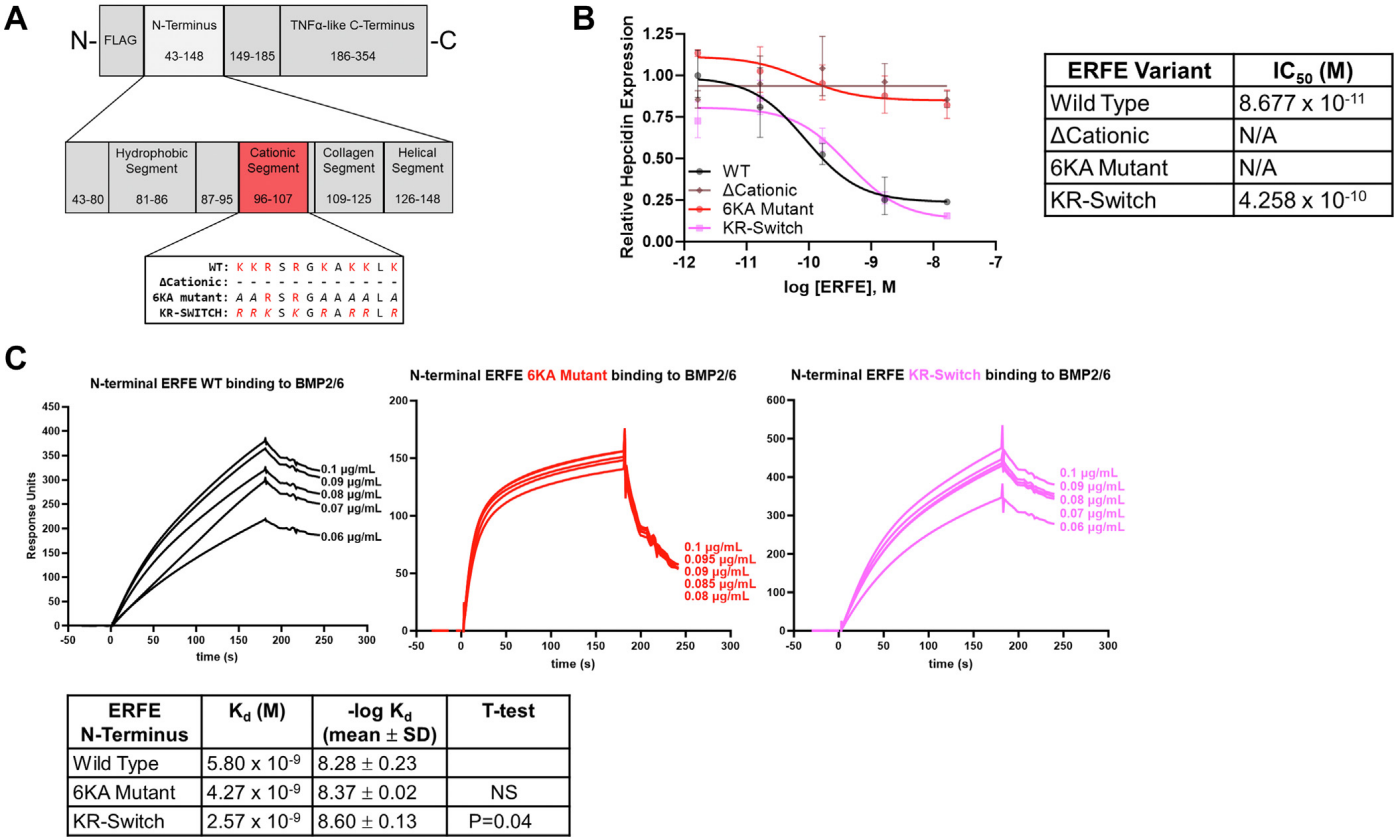
**Thecationicsegment of ERFE N terminus.***A*, diagram of three mutations in the ERFE cationic segment used for biological activity: ΔCationic (deletion of amino acids 96–107); 6KA mutant (substitution of six lysines with alanines); KR-Switch mutant (substitution of six lysines with arginines and two arginines with lysines). *B*, qPCR expression of hepcidin in Hep3B cells treated with indicated concentrations of WT, ΔCationic, 6KA mutant, and KR-Switch ERFE. Data are normalized to untreated controls, N = 3 biological replicates, statistics in Table 3. IC_50_ for hepcidin suppression by each mutant is shown on the *right*. *C*, surface plasmon resonance of N-terminal ERFE WT, 6KA mutant, and KR-Switch binding to BMP2/6, with mean K_d_ and -log K_d_ of each shown on the *right*, compared by *t* test to WT (n = 5). ERFE WT data from Figure 3 were redisplayed for ease of comparison. BMP, bone morphogenetic protein; ERFE, erythroferrone.

We next investigated the cationic segment using HEK293T cells to produce full-length ERFE lacking this region (ΔCationic) (Fig. 4*A*). Like the ΔHydrophobic mutant, the ΔCationic mutant lacks detectable bioactivity (Fig. 4*B*). Despite our SPR finding that the cationic 18-mer peptide that covers this region binds BMPs, neither of our modeling systems predicted a direct interaction of this segment with BMPs, possibly because of constraints imposed by the rest of the protein.

We next asked whether the specific sequence rather than the overall cationic character is required for ERFE bioactivity (Fig. 4*A*) and generated two additional mutants: the 6KA mutant changes the six lysines of the domain into alanines, which neutralizes much but not all of the positive charge and the KR-switch mutant turns the six lysines into arginines and the two arginines into lysines, retaining the overall charge. Similarly to ΔCationic, the 6KA mutant lacks hepcidin-suppressive activity but the bioactivity of the KR-switch mutant is comparable to that of WT ERFE (Fig. 4*B*).

To determine which, if any, of the cationic residues is required for hepcidin suppression, we performed alanine scanning through the lysines of the cationic segment. Surprisingly, no single alanine substitution had a substantial effect on function. Neutralization of three out of the six lysines was required to impair hepcidin suppression, but the choice of lysines did not appear to be important (Fig. S4*A*). Thus, overall charge interactions and not individual residues are responsible for the functional contribution of the cationic segment.

We next measured the binding of the 6KA mutant N terminus produced in bacteria to BMP2/6, 2, or 6 (Figs. 4*C* and S4, *B* and *C*). The interaction curve shows fast on- and off-rates similar to the W82A mutant which also lacks biological activity, and the KR-switch mutant binding curve is similar to that of WT (Figs. 4*C* and S4*D*).

Based on the sequence of the cationic segment, we hypothesized that this region may also function as a heparin-binding domain. Such interactions may be functionally important because the perisinusoidal space (the Space of Disse) where the contact between ERFE and BMPs occurs is particularly rich in sulfated proteoglycans (14), and sulfated proteoglycans have been strongly implicated in the regulation of iron-related BMP signaling (15, 16). Due to its large number of negatively-charged sulfo and carboxyl groups, heparin has the highest negative charge density of any known biological macromolecule (17), and heparin-binding proteins possess matching positively-charged regions like the cationic segment of ERFE to promote their interaction with heparin. We overexpressed full-length ERFE in HEK293T cells and adsorbed it to a HiTrap heparin sepharose affinity column in an FPLC instrument. Elution was performed by an NaCl gradient, and fractions were analyzed by Western blot, with heparin-bound ERFE eluting at about 0.5 M NaCl (Fig. S5), representing a medium-strength binding interaction. ΔCationic eluted at a much lower concentration of NaCl indicative of impaired heparin-binding affinity. The elution pattern of the 6KA mutant was also impaired similarly to that of ΔCationic. Surprisingly, the KR-switch mutant bound much stronger to the column than even WT. Our data therefore indicate that positive charge in the cationic segment is required for ERFE function and heparin binding.

### The collagen and helical segments make smaller contributions to ERFE bioactivity

**Figure 5:**
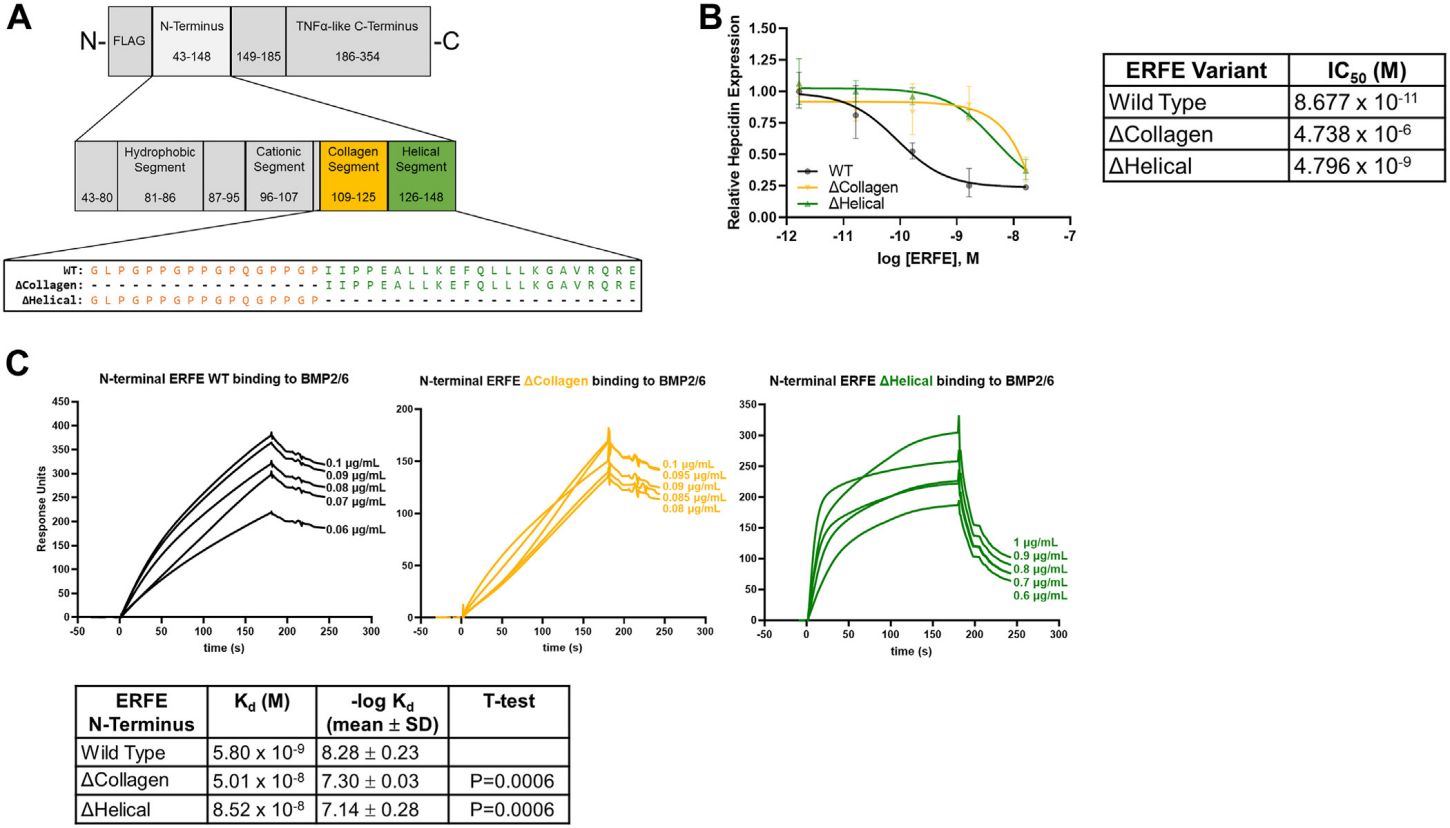
**The collagen and helical segments of ERFE N-terminus.***A*, diagram of mutations in the ERFE collagen and helical segments used for bioassays: ΔCollagen (deletion of amino acids 109–125); ΔHelical (deletion of amino acids 126–148). *B*, expression of hepcidin mRNA in Hep3B cells treated with indicated concentrations of WT, ΔCollagen, and ΔHelical ERFE. qPCR-generated data are normalized to untreated controls, N = 3 biological replicates per point, statistics in Table 1, Table 2, Table 3. IC_50_ for hepcidin suppression by each mutant is shown on the *right*, with statistics in Table 3. *C*, surface plasmon resonance sensorgrams of N-terminal ERFE WT, ΔCollagen, and ΔHelical binding to immobilized BMP2/6, with mean K_d_ and -log K_d_ of each shown on the *right*, compared by *t* test to WT (n = 5). ERFE WT data from Figure 3 were redisplayed for ease of comparison. BMP, bone morphogenetic protein; ERFE, erythroferrone.

We next generated full-length constructs in HEK293T cells in which the collagen or helical segments were deleted (Fig. 5*A*). When Hep3B cells were treated with these mutants, hepcidin mRNA expression was suppressed to near WT levels but required much higher mutant ERFE concentrations than with the WT form (Fig. 5*B* and Table 3), representing more than a 100-fold loss of potency for both ΔCollagen and ΔHelical relative to WT. The affinities of bacterial ΔCollagen and ΔHelical N terminus for BMPs are comparable to each other and lower than that of WT (Figs. 5*C*, S6, *A*–*C* and Table 1), likely contributing to the lower bioactivity of the mutants. In addition, ΔHelical exhibits comparatively very rapid dissociation rates (Table 2), and the latter could also contribute to lower bioactivity.

**Table 3:** Bioactivity assays: summary of ERFE IC_50_ (90% confidence limits) values

ERFE variant	IC_50_
WT, 43-354	89 pM (29-229)
ΔHydrophobic, 43-354 del81-86	inactive
W82A, 43-354	inactive
ΔCationic, 43-354 del96-107	inactive
6KA Mutant, 43-354	inactive
KR Switch, 43-354	426 pM (213-860)
ΔCollagen, 43-354 del109-125	10 nM (3-20)
ΔHelical, 43-354 del126-148	5 nM (2-11)

Collagen-like regions are an important feature of a diverse set of proteins including those found in plasma. Multiple proteins of the CTRP family to which ERFE belongs contain the signature Gly-X-Y repeats, frequently containing proline in the X-position and hydroxyproline in the Y-position (18, 19). The collagen motif in protein multimers forms a triple helix of these domains, although some proteins further assemble into multiples of trimers, such as the collagen hexamer of the C1q subunit of complement (20). We used AlphaFold2 to generate a model of the full-length mature ERFE trimer (Fig. S6*D*). Among the top five models, those ranked 1 and 3 show a collagen triple helix with substantial confidence (Fig. S6*F*) but the others do not. The free energies associated with the top five models are very similar which may indicate that the collagen motifs form a triple helix only transiently. We next generated a model of the ERFE trimer bound to a BMP2/6 heterodimer (Fig. S6*E*). The collagen motif does not form a triple helix in this model, possibly because the ERFE-BMP–bound state is more stable than the collagen helix. In summary, the collagen motif may make a small contribution to the forces that favor ERFE trimer formation but likely does not interact directly with BMPs.

### ERFE and BMP receptors bind BMPs in a similar manner

**Figure 6:**
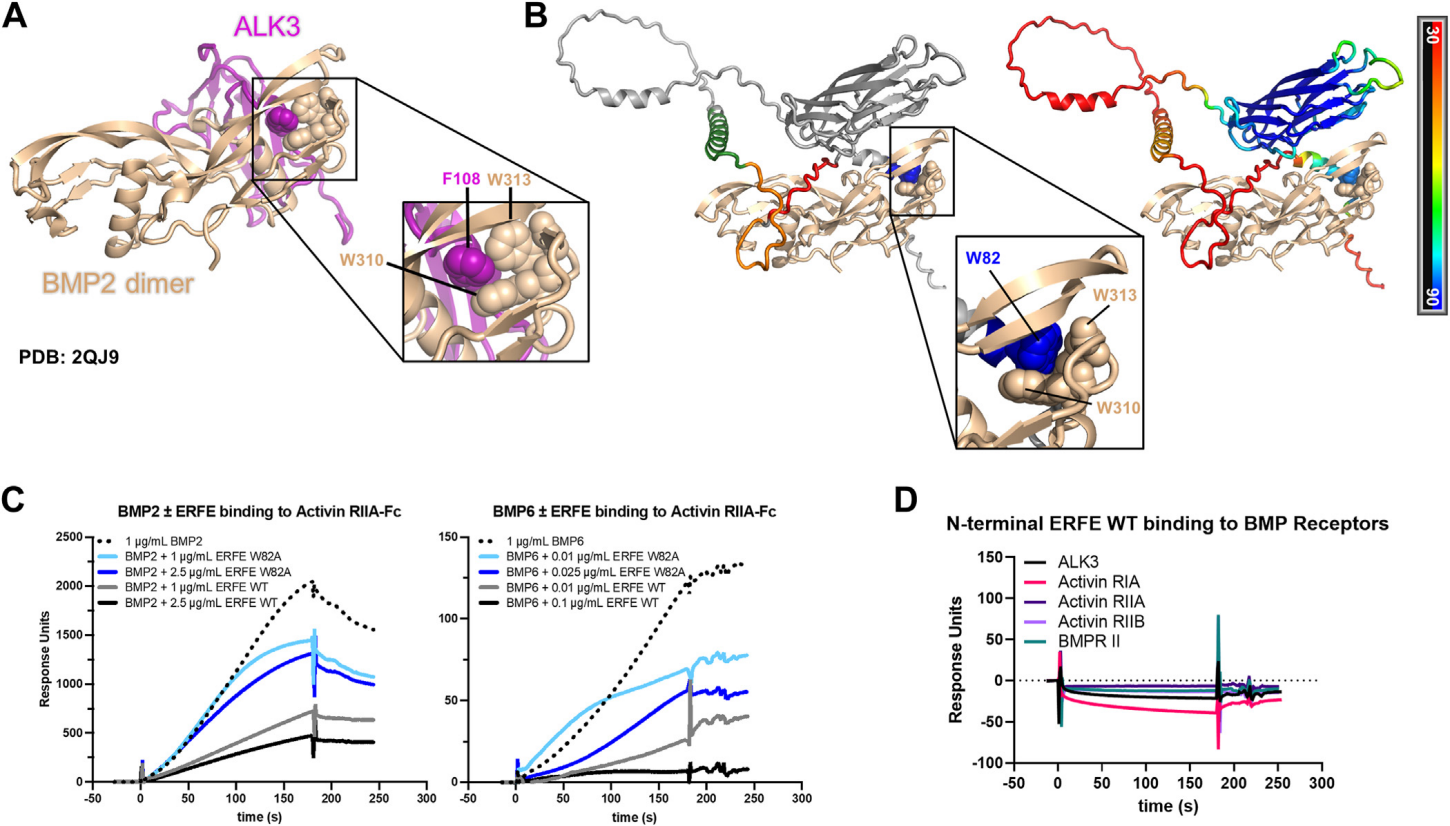
**ERFE and BMP receptors bind BMPs in a similar manner.***A*, crystallography-resolved structure of BMP2 homodimer bound to ALK3 (PDB: 2QJ9). Callout box enlarges the interaction and identifies the contacting residues. *B*, AlphaFold2 model of a BMP2 homodimer (*tan* color) bound to ERFE colored by segment (*left*) and confidence pLDDT (*right*). Callout box shows the analogous interaction that BMPs have with their receptors. *C*, surface plasmon resonance sensorgrams of competition between WT ERFE, W82A ERFE, and activin RIIA for BMP2 (*left*) and BMP6 (*right*) binding. Extracellular portions of activin RIIA were immobilized, and BMP analyte flowed over alone or mixed with WT or W82A ERFE N-terminal segments. *D*, surface plasmon resonance of the ERFE N terminus binding to extracellular portions of five BMP receptors. BMP, bone morphogenetic protein; ERFE, erythroferrone; pLDDT, predicted local distance difference test.

We sought to compare the nature of the ERFE–BMP interaction with other proteins that bind BMP molecules, the BMP receptors themselves. Crystallographic analyses have elucidated the interaction between BMPs and their receptors (21). BMP dimers form two symmetrical binding grooves, between the α-helix wrist of one molecule and the β-sheet fingers of the other. In the crystal structure of BMP2 bound to type I BMP receptor ALK3, the aromatic residue F108 of the receptor appears to associate with two tryptophan residues of BMP2 (W310 and W313) in one of the BMP-binding grooves (Fig. 6*A*). Remarkably, the same two tryptophans are shown interacting with the key W82 of ERFE in our AlphaFold2 docking model (Fig. 6*B*). Both tryptophan and phenylalanine have hydrophobic aromatic sidechains. This feature is likely crucial to binding since the ERFE W82A substitution ablated bioactivity (Fig. 3*B*) and weakened binding considerably (Fig. 3*C*). Thus, the BMP–ERFE binding interaction appears to mimic the binding of BMPs to their cognate receptors.

To test whether ERFE can directly compete with BMP receptors for binding to BMPs, we employed an SPR-based competition assay and assessed binding of both BMP2 and BMP6. In this experiment, BMPs alone or mixed with bacterial WT or W82A ERFE N-terminal segments were tested for binding to immobilized extracellular portions of BMP receptors ALK2 (Activin RIA), ALK3 (BMPR IA), Activin RIIA, Activin RIIB, and BMPRII (Figs. 6*C* and S7). WT ERFE dose-dependently interfered with BMP binding to receptors. The W82A mutant, however, competed less effectively, consistent with our bioactivity data.

The ERFE hydrophobic segment contains a second aromatic residue, F85. We speculated that ERFE may use its W82 and F85 to emulate the W-X-X-W motif in BMP molecules and bind directly to BMP receptors—a possible secondary mechanism for blocking activation of the BMP–SMAD pathway. However, the bacterial WT ERFE N-terminal segment did not bind to any of the five BMP receptors tested (Fig. 6*D*).

In addition to contact with the W-X-X-W motif, the interaction surface of BMPs with type II BMP receptors includes a contact between two receptor phenylalanines and two BMP leucines, L372 and L382 (22). Our AlphaFold2 model of BMP dimer interaction with an ERFE monomer indicates that ERFE F136 interacts with the same L372 and L382 (Fig. 2*C*) as a potential additional interaction that may help ERFE compete with type II receptors for BMP binding. However, this interaction was not visible in Alphafold2 models of ERFE multimers interacting with BMPs, which appear to be dominated by the interaction of the hydrophobic segment with the BMP groove (see further results).

### Charge interactions facilitate ERFE multimerization

**Figure 7:**
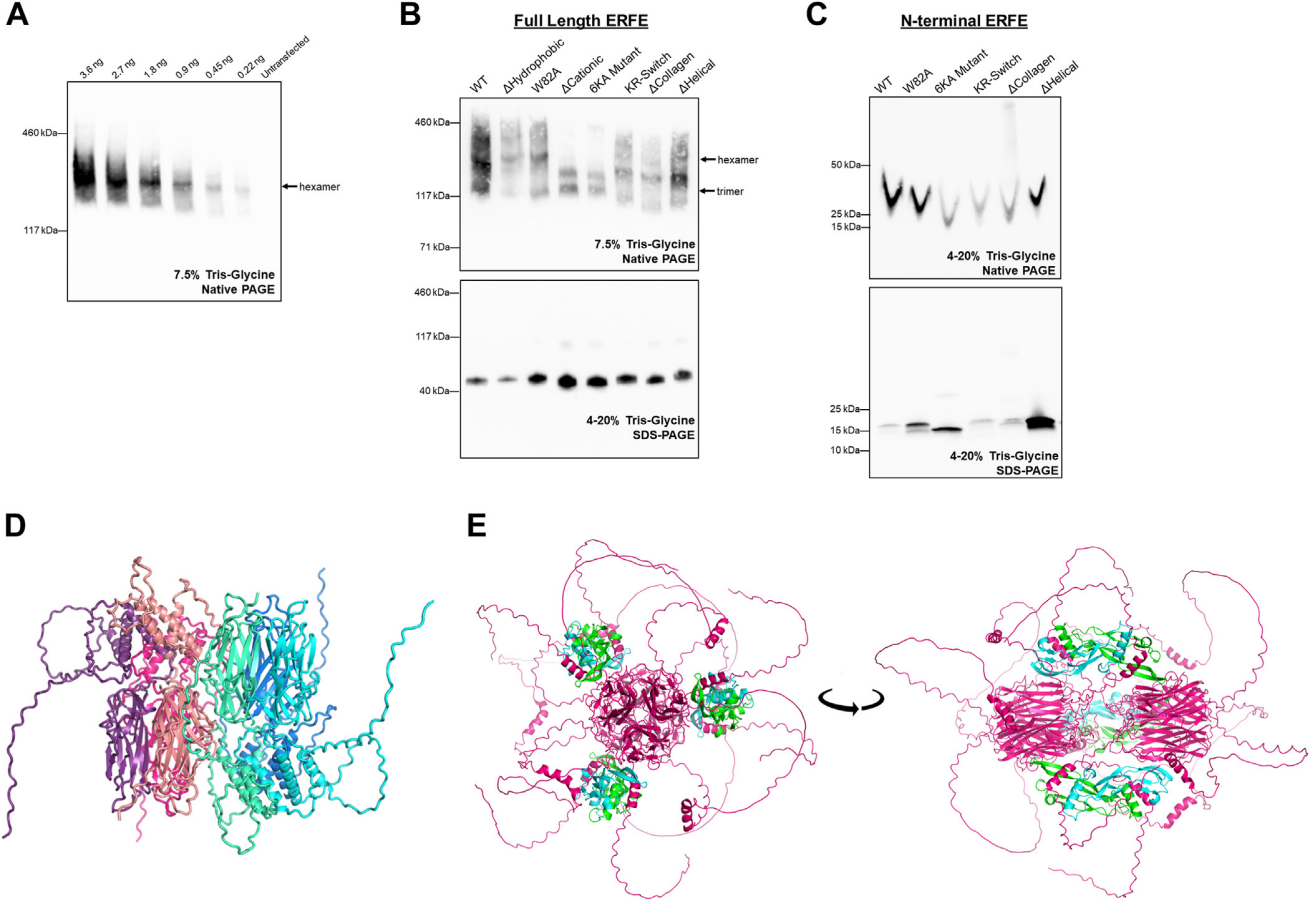
**Charge interactions facilitate ERFE multimerization.***A*, dilution series of WT ERFE containing supernatant produced in HEK293T cells revealing the hexamer as the dominant species. *B*, native (*top*) and denaturing (*bottom*) Western blots of full-length ERFE WT and mutants showing differential multimerization patterns. *C*, native (*top*) and denaturing (*bottom*) Western blots of WT and mutant N-terminal ERFE segments produced in bacteria showing a lack of multimerization. Under native running conditions, SDS which is present only in the sample dye runs unevenly through the gel at low molecular weight, causing distortion of the bands. All Western blots in (*A* and *B*) were probed with anti-FLAG HRP. Blots in (*C*) were probed with anti-ERFE. *D*, AlphaFold2 docking of hexameric human ERFE colored by chain. *E*, AlphaFold2 docking of hexameric human ERFE bound to three BMP2/6 dimers. All ERFE chains are colored *magenta*, with BMP dimers in *cyan*-*green*. BMP, bone morphogenetic protein; ERFE, erythroferrone.

Proteins in the CTRP family are known to multimerize. In the case of adiponectin, the distinct oligomeric forms have been shown to exhibit differential activities (23, 24, 25). We wondered if mutations in the ERFE N terminus could affect multimerization, and if so, if these changes might further explain the effects of mutations on ERFE bioactivity. We used native PAGE Western blotting to analyze a dilution series of ERFE-containing supernatant produced by HEK293T cells (Fig. 7*A*). At high concentrations, the ERFE band appears as a large smear between 117 kDa and 460 kDa molecular weight markers. At lower concentrations, a dominant band can be seen at an estimated mass of 260 kDa, corresponding to a hexamer.

We used this nonreducing, nondenaturing native PAGE to analyze the multimeric structures formed by ERFE mutants used in our bioassay (Fig. 7*B*, top). Most of the mutants tested formed a similar smear as WT with a prominent hexamer band. In constructs with larger deletions, this band was appropriately shifted down. Interestingly, the ΔCationic and 6KA mutants differed from the others, forming two discrete bands that appeared to be the size of a trimer. The distinct multimerization pattern in these mutants may explain why they lack biological activity despite the binding strength of the monomeric N-terminal segment 6KA mutant to BMPs being comparable to that of WT (Fig. 4, *B* and *C*). The unique banding pattern of these two mutants is confirmed to be due to differences in multimerization since the reduced, denatured forms all migrate at the same apparent size (Fig. 7*B*, bottom).

In addition to these mutations in the cationic segment, other amino acid substitutions in the N terminus have been previously shown to disrupt high order oligomer formation (10). To determine if this portion of the molecule alone is capable of forming multimers or if the C-terminal head is required, we analyzed N-terminal ERFE samples by native and denaturing PAGE (Fig. 7*C*). WT and all mutants resolve to roughly equal molecular weights in both cases, indicating that the N-terminal segment is not sufficient for multimer assembly.

Based on the estimated size of dominant ERFE multimer, we next used AlphaFold2 to model the ERFE hexamer (Fig. 7*D*). The structure resembles a dimer of trimers with interactions between the trimeric TNFα-like heads. Only the globular head region is predicted with high confidence (Fig. S8*A*). Additionally, we generated a hexamer model bound to three BMP2/6 dimers (Fig. 7*E*). Like monomeric and trimeric ERFE models of interaction with BMP2/6 (see Data S1), this model shows consistent interactions of the N-terminal hydrophobic segments of ERFE with the BMP dimers but unlike in the monomeric ERFE interaction with BMP dimers, the helical segment does not make contact here. The ERFE TNFα-like heads and hydrophobic segments that are in contact with the BMPs are predicted with the highest confidence (Fig. S8*B*).

Our data therefore indicate that at low concentrations, full-length ERFE produced by human cells *in vitro* is predominantly a hexamer, that the cationic segment of ERFE is required for this multimerization, but that the N-terminal domain is not sufficient for the multimerization. Although multimerization greatly increases the avidity of ERFE for its BMP ligand by decreasing the off-rate, multimerization is not strictly required for bioactivity since the monomeric N-terminal domain by itself suppresses hepcidin at similar molar concentrations, at least under the greatly simplified *in vitro* conditions.

### Truncating the N-terminal domain reverses the activity of ERFE

**Figure 8:**
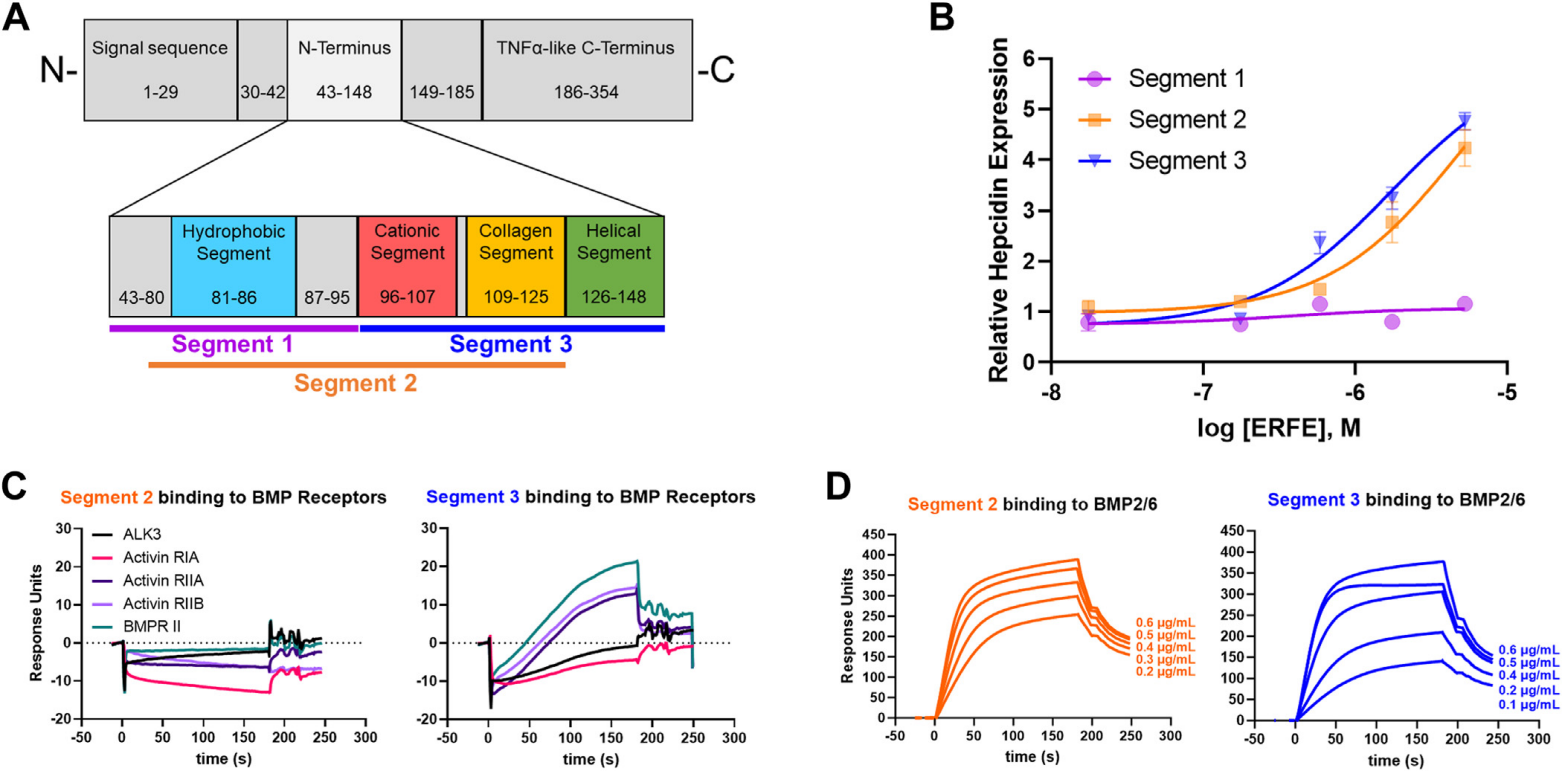
**Truncating the N-terminal domain reverses the activity of erythroferrone.***A*, diagram of generated synthetic segments of the ERFE N terminus. *B*, qPCR expression of hepcidin in Hep3B cells treated with indicated concentrations of N-terminal ERFE segments. Data are normalized to untreated controls. N = 3 biological replicates, statistics in Table 1, Table 2, Table 3. *C*, surface plasmon resonance sensorgrams of N-terminal ERFE segments 2 (*left*) and 3 (*right*) binding to BMP receptors. *D*, surface plasmon resonance sensorgram of N-terminal ERFE segments 2 (*left*) and 3 (*right*) binding to BMP2/6. BMP, bone morphogenetic protein; ERFE, erythroferrone.

In an effort to identify the minimal region necessary for ERFE function, we used peptide synthesis to produce 53-mer peptides covering the first (amino acids 43–95) and second (96–148) halves of the active N-terminal domain (segments 1 and 3, respectively), as well as segment 2 which spans the middle portion of N terminus (73–125) (Fig. 8*A*). Surprisingly, we found that treatment with either segment 2 or segment 3 not only did not suppress hepcidin but did the opposite and increased hepcidin expression in Hep3B cells. Segment 1 had neither a stimulatory nor inhibitory effect on hepcidin transcription (Fig. 8*B*). We found that the induction of hepcidin by segments 2 and 3 was caused by increased SMAD signaling, as ID1 expression, another BMP-SMAD target gene, was also increased (Fig. S9*A*). Activation of inflammation can also drive hepcidin, but this pathway was ruled out as mRNA expression of the inflammatory marker serum amyloid A1 was not increased in the treated Hep3B cells and in fact was below the threshold of our qPCR detection. Next, we tested the binding of the peptide segments to BMP receptors by SPR to rule out the possibility that these segments activate the SMAD pathway directly as receptor agonists. Like the complete N terminus, these fragments do not associate with any BMP receptor tested (Figs. 8*C* and S9*B*). We then measured the binding of these segments to BMPs. Segments 2 and 3 bound (Figs. 8*D* and S9, *C* and *D*); however, the binding was more than 1000-fold weaker than the complete N terminus and almost 30,000-fold weaker than the full-length protein (Fig. 1*E* and Tables 1 and 2). Segment 1 did not bind any BMP (not shown).

Our data therefore indicate that while the entire N-terminal domain (43–148) is required for its suppressive effect on BMP signaling, low affinity BMP binding by its segments may paradoxically facilitate BMP-SMAD signaling.

## Discussion

In this study, we confirmed that the bioactivity of ERFE depends on the N-terminal domain of the protein (9, 11) and proceeded to examine the specific structural features that determine ERFE bioactivity. We identified four evolutionarily conserved features within the N-terminal domain and designated them as hydrophobic, cationic, collagen, and helical segments. Each short linear domain demonstrated BMP-binding activity on our SPR screen, indicating possible relevance to the mechanism of action. Two independent protein docking algorithms (Alphafold2 and molecular dynamics) predicted direct contact of the W82-containing hydrophobic segment of ERFE with a specific moiety of BMP, and both algorithms also predicted interaction at the helical segment. Neither model found an interface between the cationic or collagen segments of ERFE and the BMP molecule, despite the implications of our SPR data with linear 18-mer peptides, presumably because in contrast to the linear peptides, the folded full-length protein has buried residues and greater spatial constraints.

In all of our docking models, the ERFE tryptophan W82 appears to insert between two tryptophans in BMP2, BMP6, and the BMP2/6 heterodimer—a state that may be stabilized by hydrophobic or electrostatic forces. We found the ERFE W82 to be essential to its functioning, as mutation to an alanine abolished all activity. The interaction of this key tryptophan is reminiscent of other natural BMP-binding proteins: type I BMP receptors and certain antagonists such as noggin. In the case of BMP receptors, hydrophobic and aromatic residues like tryptophan, tyrosine, and phenylalanine form critical bonds with hydrophobic BMP side chains. The exact combination of BMP and receptor residues differs depending on the pairing, and these differences strengthen specificity (26). The BMP antagonist noggin binds BMP7 by inserting a proline residue into a hydrophobic BMP pocket formed by the W-X-X-W motif (27), consistent with our ERFE findings here.

Like ERFE, noggin is a heparin-binding protein and is thought to bind heparan sulfate proteoglycans on cells, trapping BMPs at the surface. Interestingly, heparin binding by noggin appears to be independent of its BMP antagonist activity, as a mutant lacking the heparin domain continued to bind BMPs and inhibit BMP signaling in cells (28). Our measurements by SPR indicate that, like noggin, deletion or partial charge neutralization of the cationic segment had no effect on BMP-binding affinity, although the on- and off-rates differed from WT. This may be evidence that BMP binding is necessary but not sufficient for full physiological antagonist activity. Unlike with noggin, these mutations in ERFE ablated hepcidin-suppressing activity, possibly by favoring a shift to a less avid multimerization state. We found the clustered positive charge in the cationic segment but not the specific amino acid sequence to be essential for BMP binding, bioactivity, and multimer assembly since complete K-R exchange in this segment preserved all three functions. The cationic charge of this ERFE region also mediates heparin-binding activity whose function is not yet clear. Like with noggin, it may localize BMP-trapping activity to specific cell types or extracellular spaces with high concentrations of heparin-like molecules. For plasma ERFE, the main site of bioactivity is the hepatic perisinusoidal space (the Space of Disse), known to be enriched in sulfated proteoglycans (14), and sulfated proteoglycans have been strongly implicated in the regulation of iron-related BMP signaling (16). It is therefore possible that the cationic segment of ERFE allows preferential accumulation of this hormone in the proteoglycan-rich perisinusoidal space.

We noted that the deletion of the collagen segment from ERFE resulted in low yield of ERFE in the supernatant, an observation also made by others who found that retention within the cell of an immature form of ERFE is responsible for the lower yield (10). The removal of the collagen segment also decreased ERFE potency to suppress hepcidin transcription. Structurally, the collagen segment may promote trimerization by forming a triple helix, but our models suggest that this structure is not formed after the trimer binds its BMP ligand.

Similarly, the deletion of the helical segment had a relatively minor impact on ERFE bioactivity. Our modeling indicates a possible but weak interaction between BMP and ERFE at this site. Such an interaction may be more favored in ERFE monomers or isolated N-terminal segments, as the contact is not present in models of higher order multimers. In any case, its contribution to bioactivity is markedly less than that of the W82-containing hydrophobic segment. It is interesting to note that the synthetic N-terminal ERFE segment lacking the helical segment but retaining the other conserved sections (Segment 2) had greatly decreased affinity for BMPs and manifested hepcidin-inducing bioactivity. Perhaps the contributions to antagonism of some individual features are small or partially redundant in the context of the full protein but become larger when part of shorter peptides. In support of this hypothesis, segment 3 which similarly contains three out of four N-terminal segments likewise increases hepcidin transcription. When BMP-binding capacity was measured in these segments, we found that the strength of association was many-fold less than even inactive ERFE mutants. This relatively weak micromolar binding may be responsible for the switch in activity compared to full-length and N-terminal ERFE. Full-length ERFE acts as a strong trap for BMPs, with its picomolar binding avidity on par with or stronger than the binding between BMPs and receptors (29). While sometimes weaker than the WT protein, many of our mutants retain sufficiently strong binding to maintain antagonist activity. However, the binding between BMPs and segments 2 or 3 is weaker than the native interaction between BMPs and their receptors but possibly stronger than nonspecific interactions of BMPs with other molecules in the environment. Thus, these weaker-binding ERFE segments could act as agonists by chaperoning BMPs to their physiological receptors.

**Figure 9:**
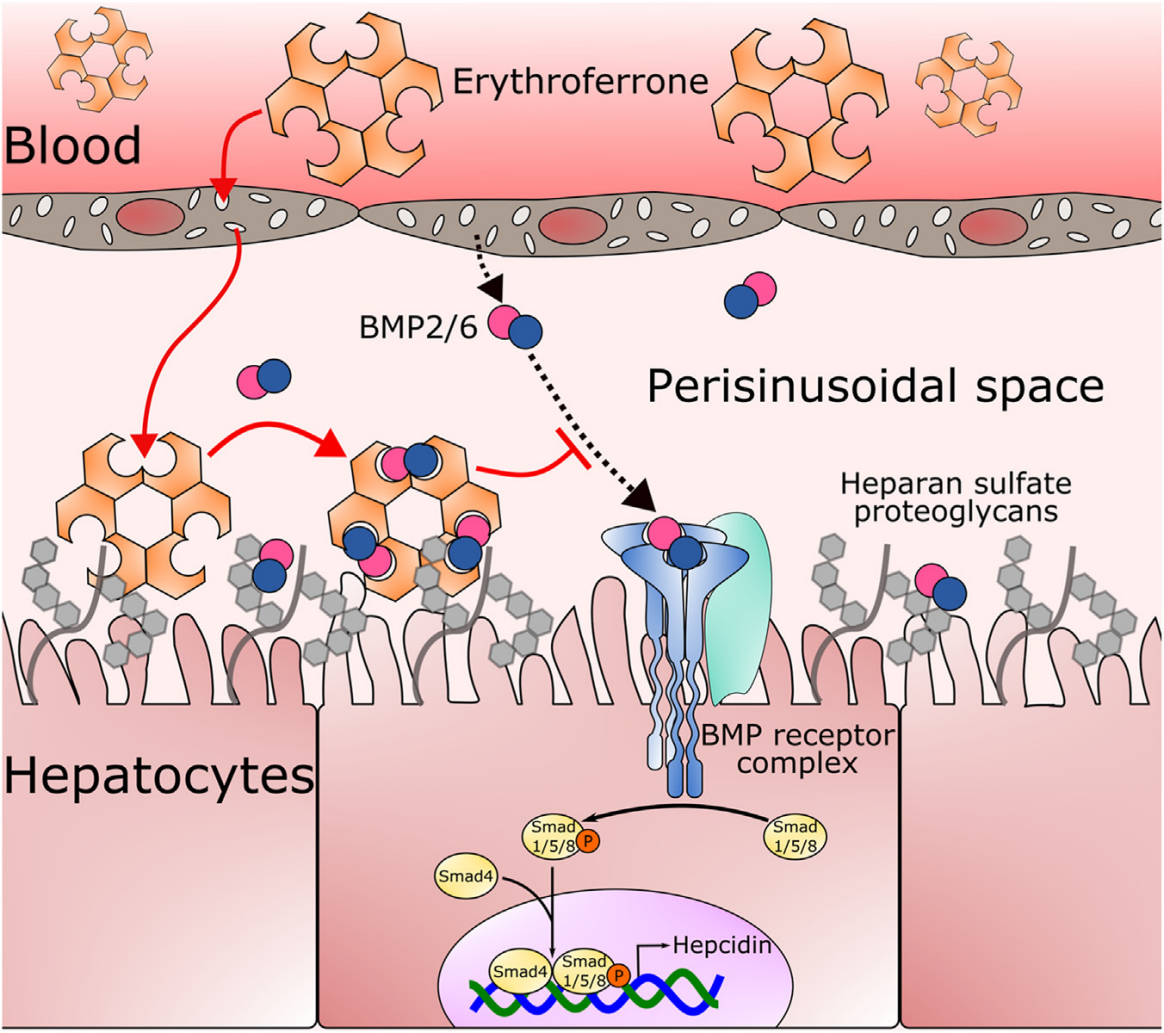
**Model of ERFE function *in vivo*.** Circulating erythroferrone diffuses into the perisinusoidal space through fenestrations in liver sinusoidal endothelial cells. Within the perisinusoidal space, ERFE may bind to heparan sulfate proteoglycans to localize to the surface of hepatocytes and bind BMP molecules. This prevents BMP binding to BMP receptors and results in lower BMP-SMAD signaling and decreased hepcidin transcription. Figure (41) modified with permission. BMP, bone morphogenetic protein; ERFE, erythroferrone.

In summary, we provide evidence for key structural features of ERFE that mediate its function (Fig. S10), allowing it to couple erythropoiesis to iron supply by inhibiting BMP signaling required for the transcription of the iron-regulatory hormone hepcidin. The ability of ERFE to bind and inactivate BMPs is mediated in large part by a key hydrophobic/aromatic interaction centered on the region around ERFE W82 interacting with the W-X-X-W motif in BMP molecules, similarly to the interaction that facilitates the binding of the ALK3 receptor to the same region of BMPs. We identified additional regions of the relatively unstructured ERFE N-terminal domain that make detectable contributions to binding BMPs (Fig. S10). We also showed that the multimeric nature of ERFE, driven both by interaction of TNFα-like C-terminal globular domains and by several features of the N-terminal domain, greatly increases the avidity of ERFE binding to BMPs by dramatically decreasing the off-rate of the binding. Finally, the heparin-binding activity mediated by the cationic segment in the N-terminal domain may help localize ERFE to its likely site of bioactivity: the perisinusoidal space rich in sulfated proteoglycans (Fig. 9). By identifying the active site and determining the structural requirements for both hepcidin inhibition and stimulation, our structure-function study of ERFE may help inform the design of new therapeutics targeting ERFE or BMPs for the treatment of iron disorders.

## Experimental procedures

### Cell culture

Hep3B cells were obtained from the American Type Culture Collection (#HB-8064) and cultured in Dulbecco’s modified Eagle’s medium (Thermo Fisher Scientific#10564029) supplemented with 10% fetal bovine serum (Genesee Scientific #25-514H) and 1% Pen/Strep (Thermo Fisher Scientific#15070063). Cells were kept in a humidified incubator at 37 °C with a 5% CO_2_ atmosphere.

### ERFE plasmid construction

For mammalian expression, FLAG-tagged full-length human ERFE (43–354) was cloned into the pcDNA3.1(+) backbone (Invitrogen #V790-20) by restriction enzyme cloning. For bacterial expression, the N terminus (43–148) of ERFE was codon-optimized for prokaryotes and synthesized as a double-strand gene block by Integrated DNA Technologies. The coding sequence was cloned into the 2M-T backbone (Addgene plasmid #29708) using In-Fusion HD (Takara #639650). The result was a TEV-cleavable N-terminal His6-MBP fusion protein. Deletions and mutations were introduced into both plasmid types by round-the-horn cloning using CloneAmp HiFi PCR premix (Takara #639298). Sequences were verified by Sanger sequencing.

### ERFE supernatant production and quantification

Supernatants containing ERFE variants were produced in HEK293T cells by transient transfection of the appropriate plasmid using Lipofectamine 3000 (Thermo Fisher Scientific#L300000) according to the manufacturer’s instructions. HEK293 and derivatives thereof have been validated to produce ERFE that is both detectable and functional upon transfection (30). One day after transfection, the cells were washed and the media was replaced with Opti-MEM (Thermo Fisher Scientific#51985034). Supernatants were collected 72 h later, aliquoted, and stored at −80 °C until use.

ERFE concentrations in supernatants were determined by Western blot for FLAG using known amounts of recombinant human His10-FLAG-BRD4 (RND Systems #SP-600) as a standard. Standards and samples were separated by SDS-PAGE and transferred to nitrocellulose membranes. Membranes were blocked in 5% w/v nonfat dry milk in TBS with 0.1% Tween-20 and incubated with monoclonal anti-FLAG M2 HRP antibody (Sigma #A8592) at a dilution of 1:20,000 in milk. Blots were visualized by chemiluminescence using the ChemiDoc imaging system and quantified using Image Lab (https://www.bio-rad.com/en-us/product/image-lab-software?ID=KRE6P5E8Z) software (Bio-Rad). The apparent size of His10-FLAG-BRD4 on the gel is about 20% larger than ERFE, so an adjustment constant of 0.81 was applied to the ERFE variants in calculating their concentrations.

### Gene expression quantification by RT-qPCR

The human hepatocyte Hep3B cell line was used because of its capacity to regulate hepcidin mRNA in response to interleukin 6 and BMPs over a broad range (4, 31), allowing us to assay for suppression as well as induction. Cells were treated overnight in a 1:1 mixture of DMEM:Opti-MEM media containing 5% serum and a dilution series of ERFE. After treatment, cells were lysed in TRIzol Reagent (Thermo Fisher Scientific#15596018), and total RNA was isolated by chloroform extraction per the manufacturer’s instructions. Five hundred nanograms of RNA was reverse-transcribed using the iScript cDNA Synthesis Kit (Bio-Rad #1708891). Quantitative real-time PCR was performed on cDNA using SsoAdvanced SYBR Green Supermix (Bio-Rad # 1725275) on the CFX Real-Time PCR Detection System (Bio-Rad). Samples were measured in technical duplicates, and target genes were normalized to HPRT. Primer sequences are provided in Table 4.

**Table 4:** Human RT-qPCR primers

Gene	Forward 5′ – 3′	Reverse 5′ – 3′
HPRT	GCCCTGGCGTCGTGATTAGT	AGCAAGACGTTCAGTCCTGTC
HAMP	GACCAGTGGCTCTGTTTTCC	AGATGGGGAAGTGGGTGTCT
ID1	TCAACGGCGAGATCAGCG	CTTCAGCGACACAAGATGCG
SAA1	GAGCACACCAAGGAGTGATTT	GAAGCTTCATGGTGCTCTCT

### Expression and purification of N-terminal ERFE for SPR

Expression plasmids containing MBP-fusion proteins were transformed into Rosetta2(DE3)pLysS cells and plated onto ampicillin+chloramphenicol LB-agar plates. Starter cultures of 50 ml were inoculated with single colonies and incubated overnight at 200 rpm at 37 °C. In the morning, 1 L of LB with antibiotics was inoculated with the starter culture and allowed to grow to A_600_ = 0.6 to 0.8 (mid-log phase). IPTG (GoldBio #I2481) was added to a final concentration of 1 mM, and the cultures were incubated for an additional 4 to 6 h. Cultures were spun down, the pellets were washed with PBS, and stored at −80 °C until purification.

For purification, cell pellets were freeze-thawed three times between 37 °C and −80 °C and resuspend in 5× volume bacterial lysis buffer (PBS + 0.1% Tween-20 + 1% Trition X). Samples were homogenized by Dounce and then sonicated on ice 10 s on and 10 s off for 1 min. Lysates were then cleared at 25,000*g* for 1 h at 4 °C. Lysate pH was adjusted to pH 8 with NaOH, and imidazole was added to a final concentration of 10 mM. Lysates were incubated with HisPur Ni-NTA (Thermo Fisher Scientific#88221), washed, and eluted with PBS-based imidazole buffers. Purification was monitored by Imperial Protein Stain (Thermo Fisher Scientific#24615) on an SDS-PAGE gel of samples taken throughout the process. Appropriate fractions were pooled and concentrated in 30K Spin-X UF molecular weight cutoff concentrators (Corning #431484) overnight at 4 °C. The next day, samples were incubated with homemade TEV protease (expressed and purified from Addgene #8827) (32) and reaction buffer (50 mM Tris–HCl, 0.5 mM EDTA, 1 mM DTT) at 4 °C for 72 h. Cleaved His-MBP tag and added TEV protease were both removed *via* reverse IMAC, leaving the purified ERFE in the flowthrough. ERFE was quantified by Imperial Protein Stain on an SDS-PAGE gel and stored in 40% ethylene glycol at −20 °C.

### N-terminal ERFE small and large peptide generation

18-mer peptides spanning the N terminus of human ERFE used for SPR along with 53-mer peptides (Segments 1–3) were all synthesized by GenScript at ≥70% purity. All peptides were resuspended to 10 mg/ml with sterile H_2_O and frozen at −20 °C. Segments 1 to 3 were thawed and briefly sonicated in a sonicating water bath before use.

### Determination of binding affinities by SPR

Proteins were immobilized on CM5 chips using a Biacore T200 instrument by amine coupling per the manufacturer’s instructions, using reagents (N-hydroxysuccinimide, 1-ethyl-3-(3-aminopropyl)carbodiimide hydrochloride and ethanolamine hydrochloride) purchased from Cytiva and 50 μg/ml of BMP2, BMP6, or BMP2/6. The analyte buffer, HBS-EP, contained 0.15 M NaCl, 3 mM EDTA, 0.005% (v/v) surfactant P20, and 0.01 M Hepes (pH 7.4). Binding was monitored at 1-s intervals for 3 min with an analyte flow rate of 50 μl/min. Dissociation was monitored at 1-s intervals for 2 to 7 min. The sensor chips were regenerated by washing with 10 mM HCl. For the SPR competition assay, Activin RIIA-Fc (#340-RC2-100), BMPRIA/ALK3-Fc (#2406-BR-100), BMPRII-Fc (#811-BR-100), Activin RIA/ALK2-Fc (#637-AR-100), Activin RIIB-Fc (#339-RB-100/CF), human IgG1-Fc for control (#110-HG-100), BMP2/6 (#7145-BP-010/CF), BMP2 (#355-BM-010/CF), and BMP6 (507-BP-020/CF) were all obtained from RND Systems. Data analysis was performed using BIAevaluation 4.1 (https://www.cytivalifesciences.com/en/us/support/software/biacore-downloads#) software from Biacore. The equilibrium dissociation constant was calculated as K_D_ = k_off_/k_on_ at five different ligand concentrations and statistics calculated using GraphPad Prism (https://www.graphpad.com/features).

### ERFE multimerization analysis

Samples were mixed with 6× nonreducing SDS-loading buffer (Boston BioProducts #BP-111NR) and separated on 7.5% tris-glycine gels (Bio-Rad #4561024). Electrophoresis and transfer were both carried out using a tris-glycine native buffer lacking both SDS and methanol (Thermo Fisher Scientific#LC2672). After transfer, the nitrocellulose membranes were blocked, probed, and developed as described above. For the untagged N-terminal segments, we used monoclonal rabbit primary antibody against ERFE mAb #9 at 1:1000 that we previously developed for the detection of human serum ERFE by ELISA (30). HRP-conjugated anti-rabbit IgG (Cell Signaling Technology #7074) was used at 1:5000 as secondary.

### ERFE modeling and docking by AlphaFold2

To model ERFE structures with or without interacting BMP, we used ColabFold v1.5.2: Alphafold2-multimer using MMseqs2 software on Google Colab Pro server, as described (33, 34). The template mode was none, the number of recycles was 20, the number of seeds (num_seeds) was 1, and the sequence alignment mode (msa_mode) was mmseqs_uniref_env. Amber relaxation was not used. The top ranking PDB file was displayed using Pymol 2.5.3 (Schrodinger, LLC). BMP numbering is based on the full, unprocessed sequence including pro-protein elements. UniProt accession numbers: BMP2 (P12643), BMP6 (P22004).

### Prediction of ERFE molecular complexes using neural network–assisted docking and structural refinement with molecular dynamics (Charmm)

The initial structures for the protein complexes were determined using artificial intelligence, neural network–machine learning based approach with AlphaFold (34, 35). The AlphaFold program was run through the Chimera X (version 1.5) molecular modeling system environment (36) available at https://www.cgl.ucsf.edu/chimera/docs/relnotes.html. These initial predicted protein complex structures were then refined using molecular dynamics to provide a more accurate representation of the complex in aqueous environments.

The protein complex was uploaded to the Charmm Solution Builder (http://www.charmm-gui.org) (37, 38, 39) and placed into a rectangular 120.0 × 120.0 × 120.0 Å simulation box with the protein complex positioned at 10 Å from the edge of the box. The system then was hydrated with TIP3 waters (40), and potassium and chloride ions were then added to render the ensemble electrically neutral. The simulation box was then downloaded from the Charmm GUI website server using the Gromacs simulation option to set up the system for the equilibration and production runs. Molecular dynamic simulations were carried out using the Charmm 36m all atom force field implementation for aqueous solvents and proteins in the Gromacs (Version 2022.4) environment (http://www.gromacs.org). The system was first minimized using a steepest descent strategy followed by a six-step equilibration process at 311 K for a total of 500 ns. This included both NVT (constant number, volume, temperature) and NPT (constant number, pressure, temperature) equilibration phases to allow water molecules to reorient around the lipid headgroups and any exposed parts of the peptide, as well as permitting lipids to optimize their orientation around the peptide. Equilibration protocols employed a Particle Mesh Ewald strategy for Coulombic long-range interactions and Berendsen temperature coupling. A Berendsen strategy was also used for pressure coupling in a semi-isotropic mode to emulate bilayer motion. After equilibration, the system was subjected to a dynamics production run at the same temperature using the Nose-Hoover protocol and pressure (Parrinello-Rahman) values used in the pre-run steps The Verlet cut-off scheme was employed for all minimization, equilibration, and production steps. Detailed script and parameter files for this solution simulation were generated by the Charmm-GUI website: (http://www.charmm-gui.org). The output of the production run simulations was analyzed with the Gromacs suite of analysis tools. The coordinates for the lowest energy conformers of the protein complexes as well as detailed dynamics refinement protocols are in the Supplemental materials.

### Statistical analysis

Gene expression data are presented as XY plots with symbols indicating the geometric mean, and the error bars indicating geometric SD. SPR data are presented as single-point XY plots with points recorded at 1-s intervals. Table 2 contains log association constants log Ka analyzed from n = 5 plasmon resonance curves using Biacore statistical package and presented as mean ± SD of log Ka. SDs are shown for log K_a_ because K_a_ varies over several orders of magnitude. Table 3 contains means and 90% confidence limits calculated from sigmoid nonlinear fits to dose response data (n = 3 biological replicates per data point). Statistical analysis was performed using GraphPad Prism.

## Data availability

All data are contained within the article and its supporting information.

## Supporting information

This article contains supporting information.

## Conflict of interest

T. G. and E. N. are shareholders and scientific advisors of Intrinsic LifeSciences and consultants for Ionis Pharmaceuticals, Disc Medicine, Silence Therapeutics Chugai, and Vifor. E. N. is a consultant for Protagonist, and T. G. is a consultant for Akebia, Dexcel, and Avidity Bio. Other authors declare that they have no conflicts of interest with the contents of this article.

## Supporting information

PDB Supplement

Supplemental Figures

Supplemental Structural Data
