# Supplementary material for: Characterization of erythroferrone structural domains relevant to its iron-regulatory function
Source: J Biol Chem. 2023 Oct 20;299(12):105374. doi: 10.1016/j.jbc.2023.105374 (PMC10692919; doi:10.1016/j.jbc.2023.105374)
Supplement: PDB Supplement [file mmc1.zip › PDB Supplement/Supplemental Structural Data.docx]

**Supplemental Data**

**PDB files of structures shown in figures**

Figure 1B and Figure 2B: [Erfe monomer.pdb](PDB%20Supplement/Erfe%20monomer%20with%20signal%20sequence%20Alphafold%20catalog.pdb)

Figure 2C: [ErfeBMP2BMP6_f5f65.pdb](file:///C:\Users\tganz.AD\Google%20Drive\Manuscripts\Danny%20Srole%202023\PDB%20Supplement\ErfeBMP2BMP6_f5f65_unrelaxed_rank_001_alphafold2_multimer_v3_model_5_seed_000.pdb)

Figure 2D: [erfebmp2_10ns.pdb](PDB%20Supplement/erfebmp2_10ns.pdb)

Figure S-2C: [ErfeBMP2BMP6_f5f65.pdb](file:///C:\Users\tganz.AD\Google%20Drive\Manuscripts\Danny%20Srole%202023\PDB%20Supplement\ErfeBMP2BMP6_f5f65_unrelaxed_rank_001_alphafold2_multimer_v3_model_5_seed_000.pdb)

Figure S-2D: [ErfeBMP2BMP2_ad398.pdb](file:///C:\Users\tganz.AD\Google%20Drive\Manuscripts\Danny%20Srole%202023\PDB%20Supplement\ErfeBMP2BMP2_ad398_unrelaxed_rank_001_alphafold2_multimer_v3_model_5_seed_000.pdb)

Figure S-2E: [ERFEBMP6BMP6_acb11.pdb](file:///C:\Users\tganz.AD\Google%20Drive\Manuscripts\Danny%20Srole%202023\PDB%20Supplement\ERFEBMP6BMP6_acb11_unrelaxed_rank_001_alphafold2_multimer_v3_model_4_seed_000.pdb)

Figure S-6D: [Erfetrimer_20c31.pdb](file:///C:\Users\tganz.AD\Google%20Drive\Manuscripts\Danny%20Srole%202023\PDB%20Supplement\Erfetrimer_20c31_unrelaxed_rank_001_alphafold2_multimer_v3_model_5_seed_000.pdb)

Figure S-6E: [ErfetrimerBMP2BMP6_33fdd.pdb](file:///C:\Users\tganz.AD\Google%20Drive\Manuscripts\Danny%20Srole%202023\PDB%20Supplement\ErfetrimerBMP2BMP6_33fdd_unrelaxed_rank_001_alphafold2_multimer_v3_model_4_seed_000.pdb)

Figure 6B: [ErfeBMP2BMP2_ad398.pdb](file:///C:\Users\tganz.AD\Google%20Drive\Manuscripts\Danny%20Srole%202023\PDB%20Supplement\ErfeBMP2BMP2_ad398_unrelaxed_rank_001_alphafold2_multimer_v3_model_5_seed_000.pdb)

Figure 7D: [ERFEhexamer_83bb7.pdb](file:///C:\Users\tganz.AD\Google%20Drive\Manuscripts\Danny%20Srole%202023\PDB%20Supplement\ERFEhexamer_83bb7_unrelaxed_rank_001_alphafold2_multimer_v3_model_4_seed_000.pdb)

Figure 7E: [ERFEhexamerBMP2BMP6x3_90ba3.pdb](file:///C:\Users\tganz.AD\Google%20Drive\Manuscripts\Danny%20Srole%202023\PDB%20Supplement\ERFEhexamerBMP2BMP6x3_90ba3_unrelaxed_rank_001_alphafold2_multimer_v3_model_3_seed_000.pdb)
