# Supplementary material for: Characterization of erythroferrone structural domains relevant to its iron-regulatory function
Source: J Biol Chem. 2023 Oct 20;299(12):105374. doi: 10.1016/j.jbc.2023.105374 (PMC10692919; doi:10.1016/j.jbc.2023.105374)
Supplement: Supplemental Data 1 [file mmc2.docx]

2023-05-15 21:33:11,788 Query 1/1: ErfeBMP2BMP2_ad398 (length 518)

2023-05-15 23:48:23,625 rank_001_alphafold2_multimer_v3_model_5_seed_000 pLDDT=69.5 pTM=0.473 ipTM=0.754

Predicted alignment error


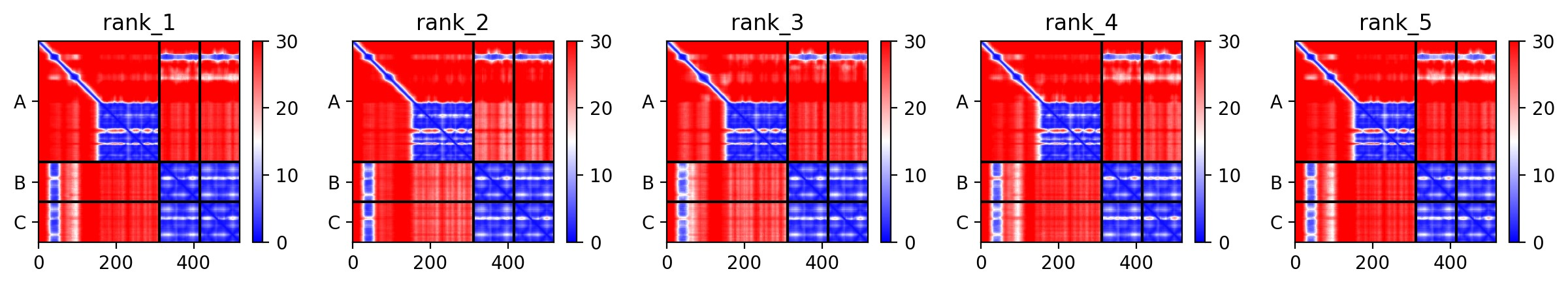


A = ERFE B = BMP2 C = BMP2

2023-05-07 02:14:27,748 Query 1/1: ErfeBMP2BMP6_f5f65 (length 518)

2023-05-07 02:30:37,696 rank_001_alphafold2_multimer_v3_model_5_seed_000 pLDDT=70.2 pTM=0.497 ipTM=0.825

Predicted alignment error


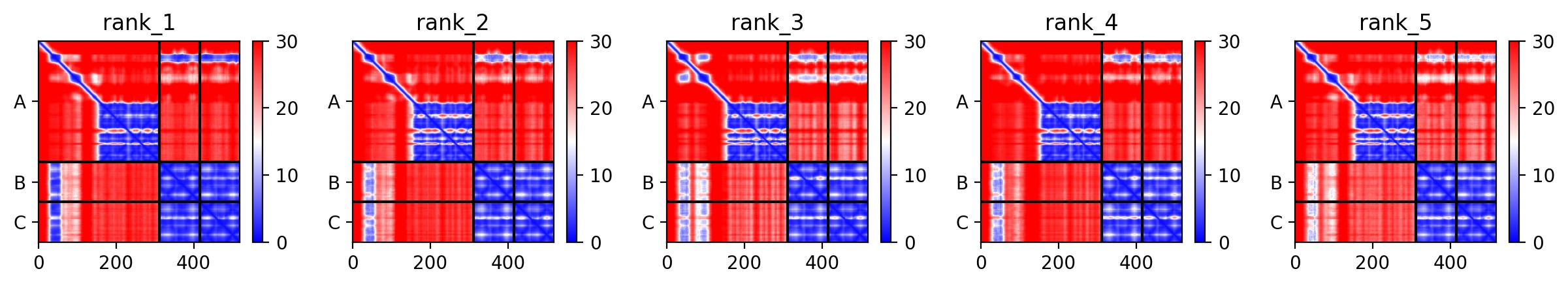


A= ERFE B = BMP2 C = BMP6

2023-07-11 05:08:13,991 Query 1/1: ERFEBMP6BMP6_acb11 (length 518)

2023-07-11 07:22:28,827 rank_001_alphafold2_multimer_v3_model_4_seed_000 pLDDT=71.6 pTM=0.476 ipTM=0.808

Predicted alignment error


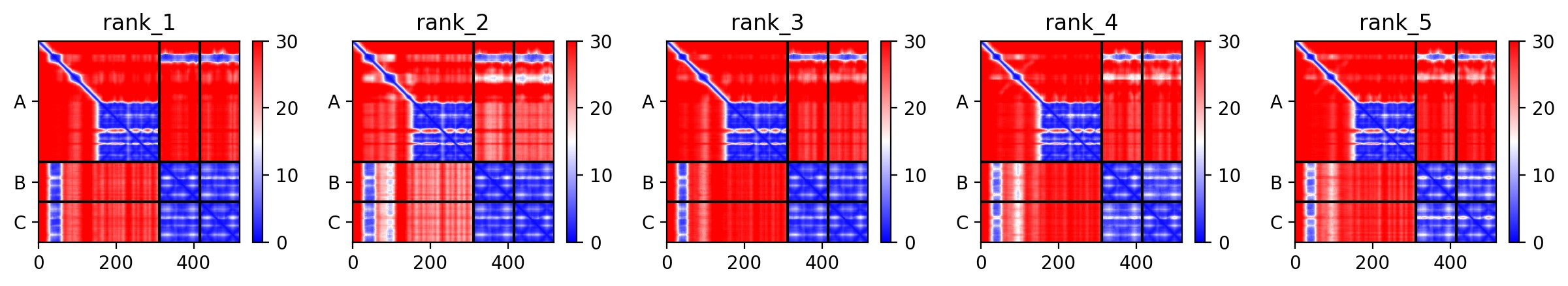


A= ERFE B = BMP6 C = BMP6

2023-07-06 23:35:40,663 Query 1/1: ERFEhexamer_83bb7 (length 1872)

2023-07-07 02:42:49,200 rank_001_alphafold2_multimer_v3_model_4_seed_000 pLDDT=52.8 pTM=0.351 ipTM=0.297

Predicted alignment error


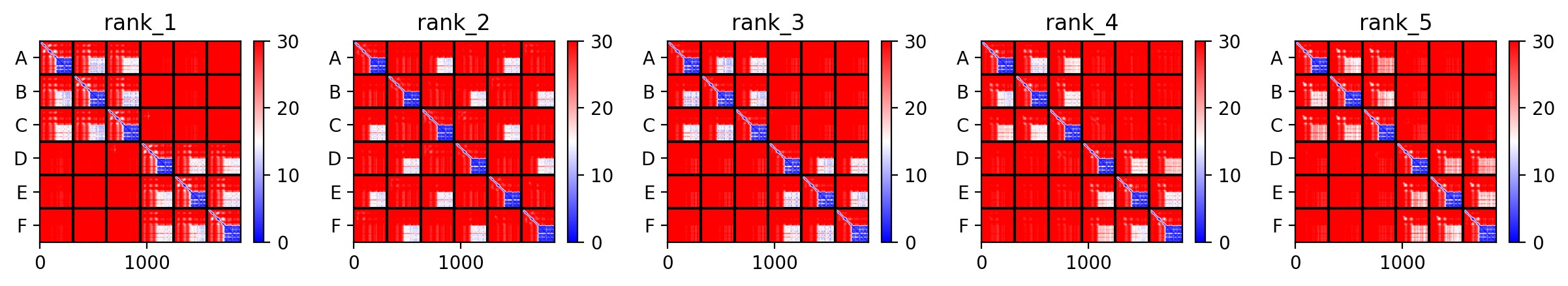


A,B,C,D,E,F = ERFE

2023-07-07 20:14:31,193 Query 1/1: ERFEhexamerBMP2BMP6x3_90ba3 (length 2490)

2023-07-08 04:12:39,582 rank_001_alphafold2_multimer_v3_model_3_seed_000 pLDDT=61.9 pTM=0.543 ipTM=0.529

Predicted alignment error


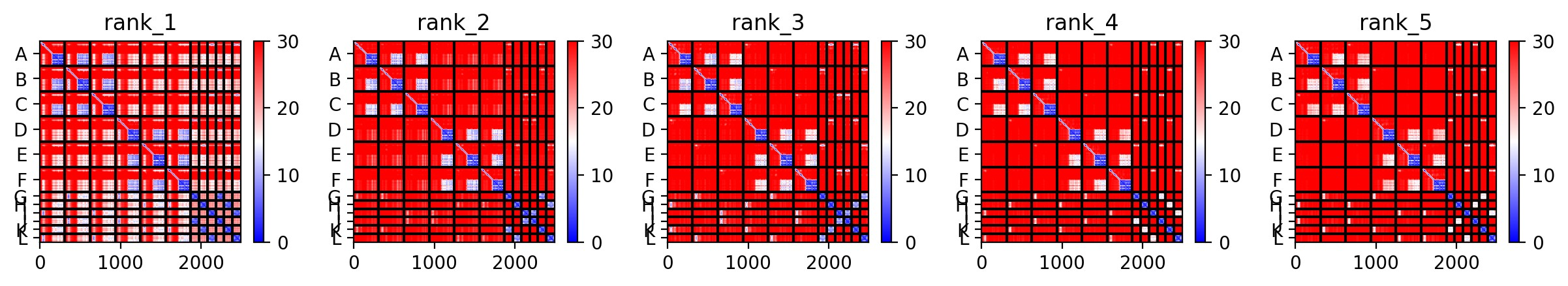


A,B,C,D,E,F = ERFE G,I,K =BMP2 H,J,I = BMP6

2023-05-05 06:06:02,773 Query 1/1: Erfetrimer_20c31 (length 936)

2023-05-05 07:14:35,207 rank_001_alphafold2_multimer_v3_model_5_seed_000 pLDDT=61.5 pTM=0.606 ipTM=0.602

Predicted alignment error


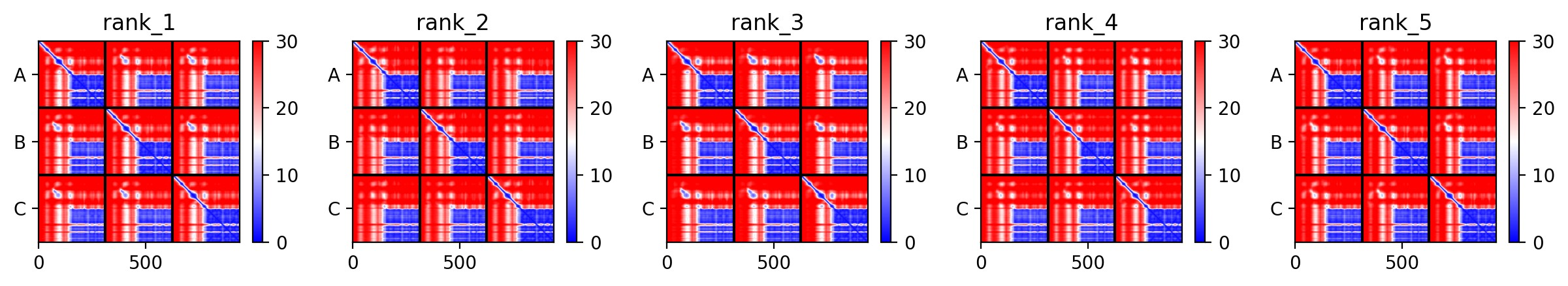


A,B,C = ERFE

2023-05-05 16:18:32,339 Query 1/1: ErfetrimerBMP2BMP6_33fdd (length 1142)

2023-05-05 17:34:13,869 rank_001_alphafold2_multimer_v3_model_4_seed_000 pLDDT=62.3 pTM=0.49 ipTM=0.455

Predicted alignment error


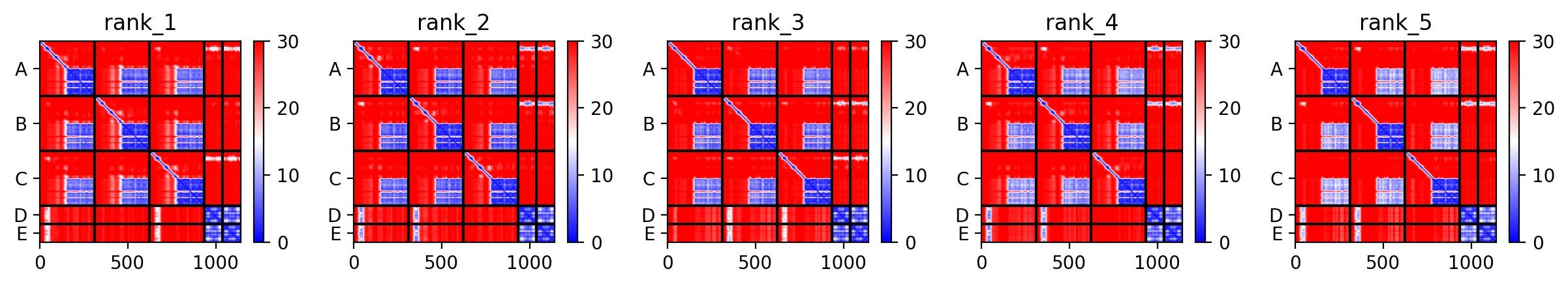


A,B,C = ERFE D = BMP2 E = BMP6
