# Supplementary material for: Characterization of erythroferrone structural domains relevant to its iron-regulatory function
Source: J Biol Chem. 2023 Oct 20;299(12):105374. doi: 10.1016/j.jbc.2023.105374 (PMC10692919; doi:10.1016/j.jbc.2023.105374)
Supplement: Supplemental Figures [file mmc3.pdf]

Supplemental Figures

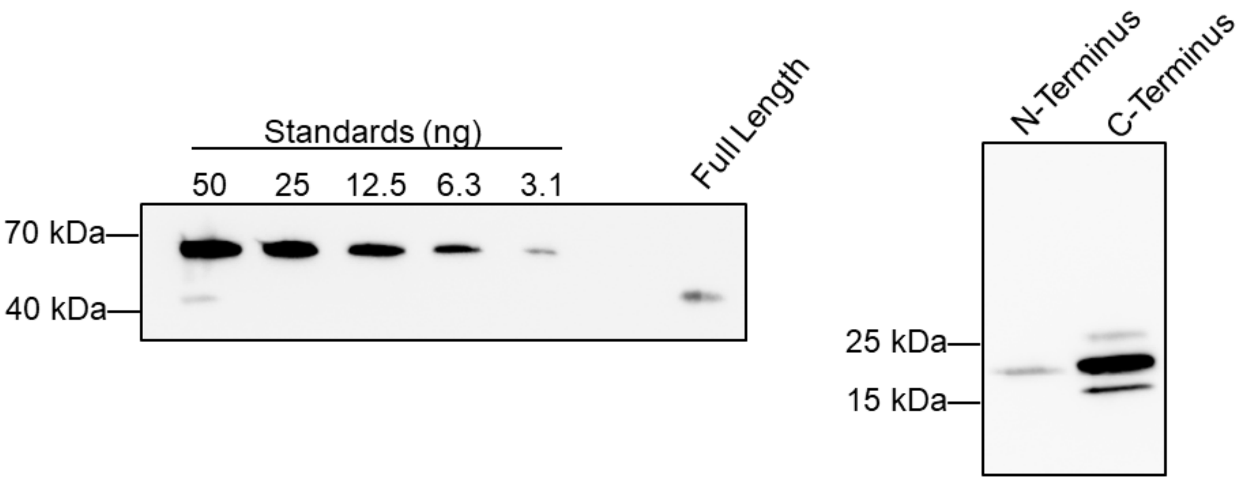

Figure S-1: Quantitative Western blots of supernatants from HEK293T cells expressing full-length, N-terminal, and C-terminal ERFE. All samples were analyzed alongside a range of amounts of FLAG-tagged standard (recombinant human His10-FLAG-BRD4, RnD Systems #SP-600) using 4-20% reducing tris-glycine PAGE. Blots were probed with anti-FLAG HRP. The apparent size of His10-FLAG-BRD4 on the gel is about 20% larger than ERFE, so an adjustment constant of 0.81 was applied to the ERFE variants in calculating their concentrations.

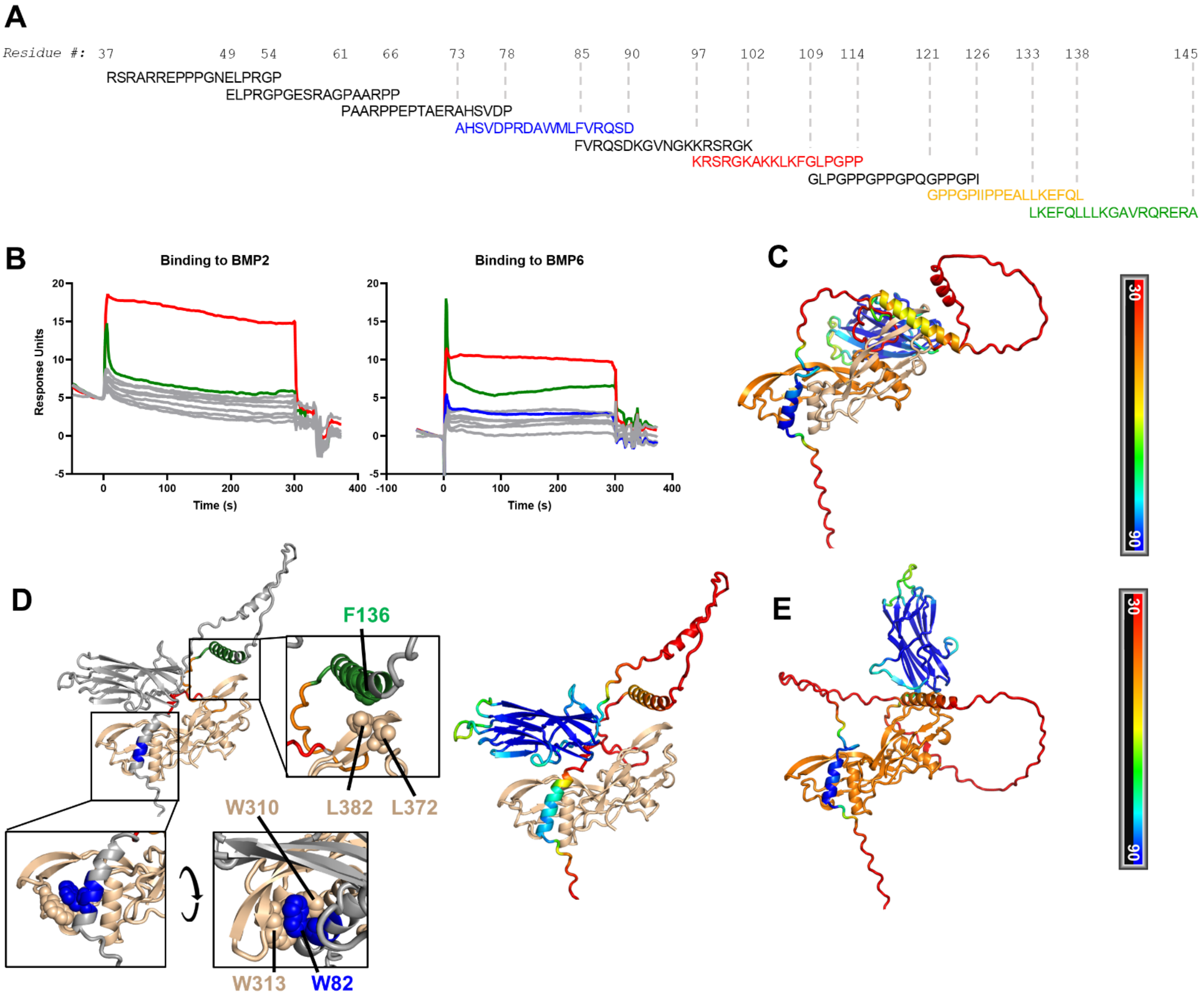

Figure S-2: (A) Diagram of overlapping synthetic 18-mer peptides used to scan the ERFE N-terminus for BMP-binding interactions. Color coding corresponds to the structural segments outlined in Figure 2B. (B) Surface plasmon resonance sensorgrams of 18-mer peptides binding to BMP2 and BMP6, with a 180 sec injection phase (end indicated by vertical dashed line) and subsequent buffer wash. (C) AlphaFold2 model of ERFE bound to a BMP2/6 heterodimer colored by confidence pLDDT (blue = high confidence). (D) AlphaFold2 model of ERFE bound to a BMP2 homodimer colored by structural segment (left) and confidence (right). (E) Alphafold2 model of ERFE bound to a BMP6 homodimer colored by confidence. (C-E) BMP2 is colored beige and BMP6 orange.

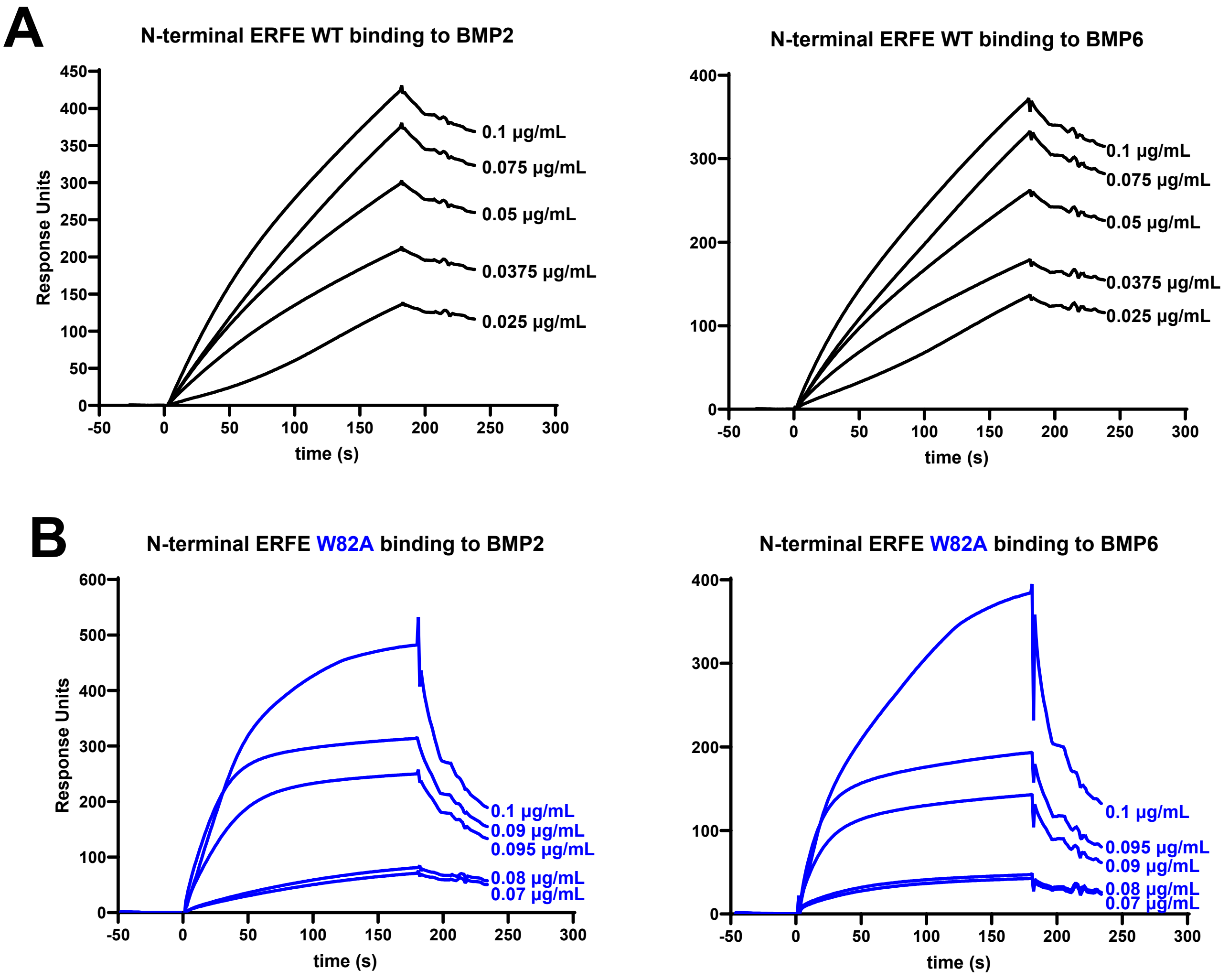

Figure S-3: Surface plasmon resonance sensorgrams of N-terminal ERFE (A) WT and (B) W82A binding to BMP2 and BMP6.

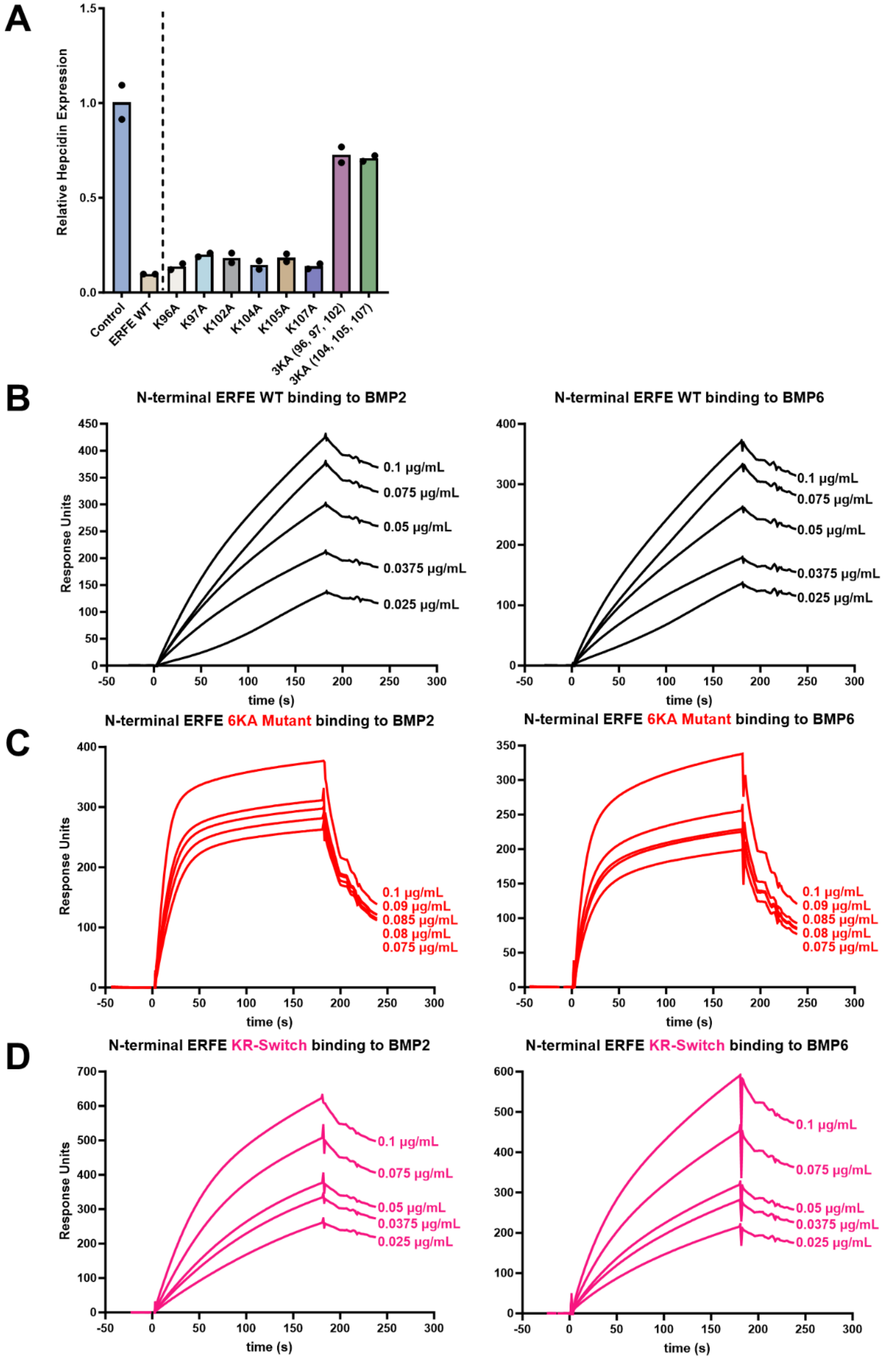

Figure S-4: (A) Expression of hepcidin mRNA in Hep3B cells treated with high concentrations of each indicated ERFE variant (10^{-8} M). The qPCR generated data are normalized to untreated controls. N = 2 biological replicates and their mean are shown. Surface plasmon resonance sensorgrams of N-terminal ERFE (B) WT redisplayed for convenience from Figure S3, (C) 6KA Mutant, and (D) KR-Switch binding to immobilized BMP2 and BMP6.

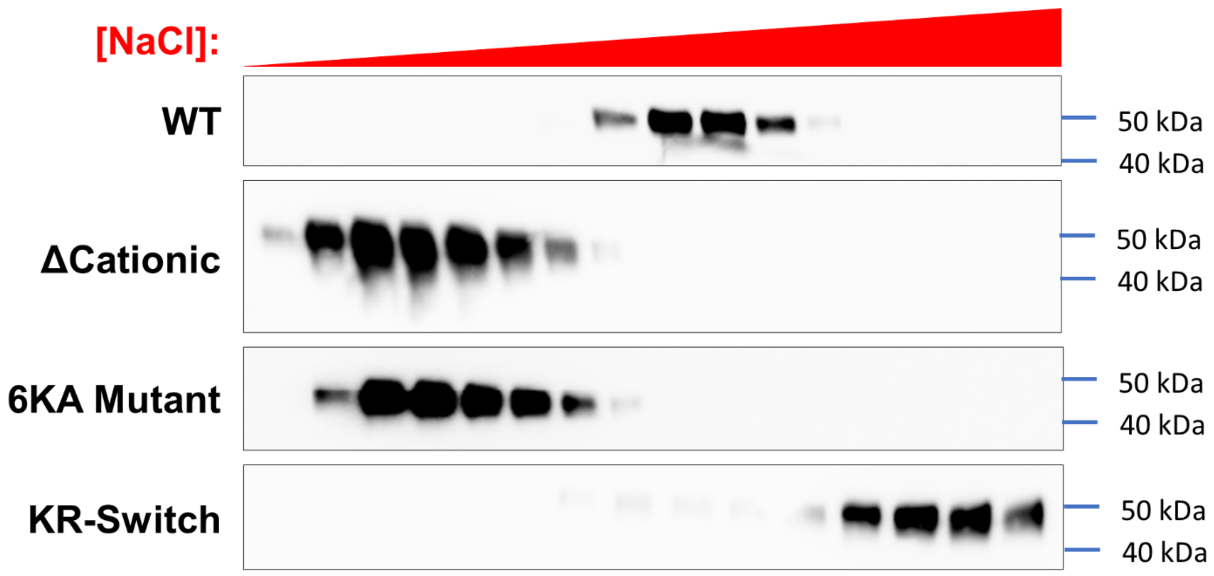

Figure S-5: WT, ΔCationic, 6KA Mutant, and KR-Switch ERFE fractions were bound to a heparin sulfate column and eluted with increasing concentrations of NaCl. Western blots of eluted fractions are shown. ERFE was detected using anti-FLAG HRP antibody.

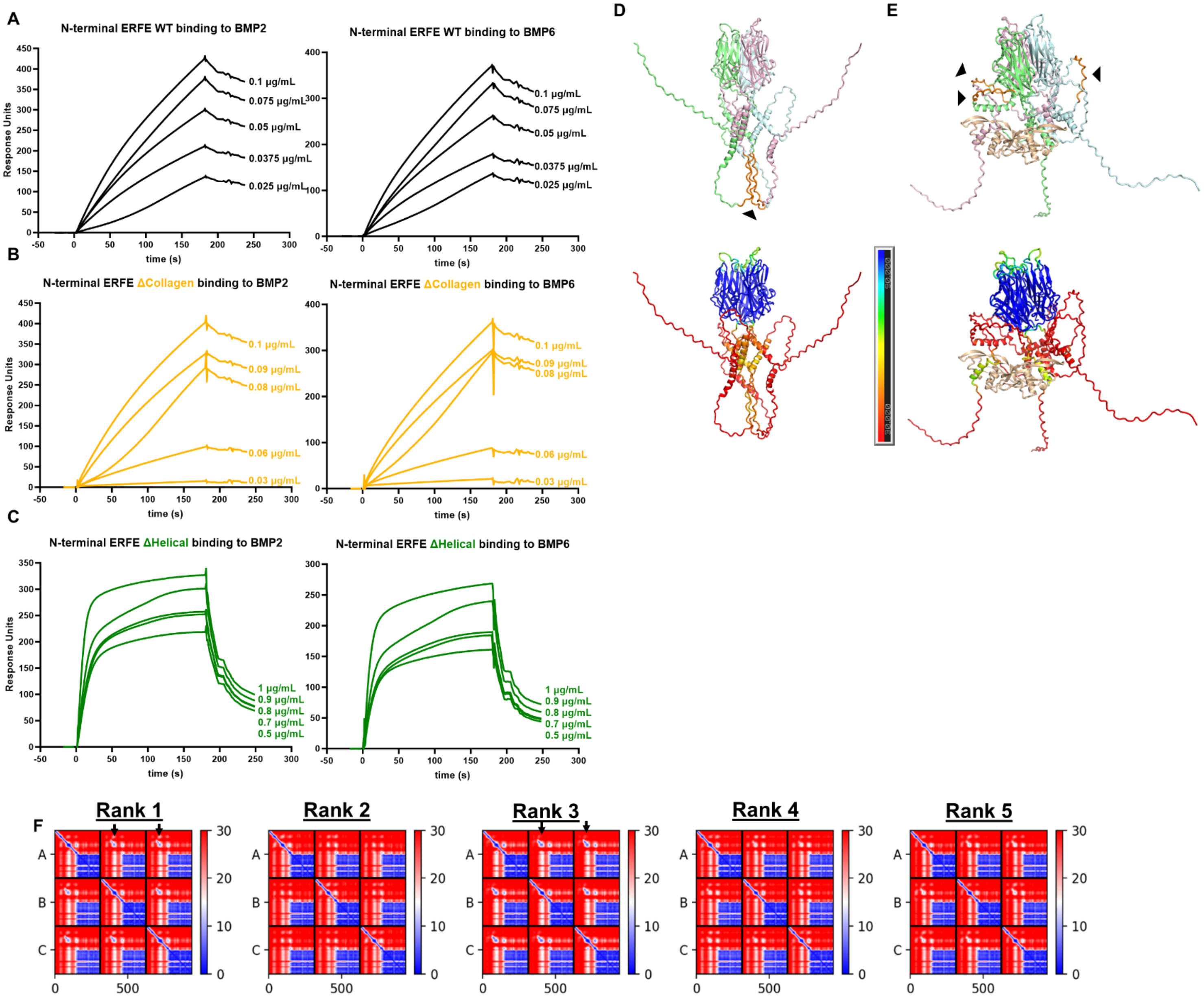

Figure S-6: Surface plasmon resonance sensorgrams of N-terminal ERFE (A) WT redisplayed for convenience from Figure S3, (B) ΔCollagen, and (C) ΔHelical binding to immobilized BMP2 and BMP6. (D) AlphaFold2 rank 1 model of ERFE homotrimer colored by chain (top). Collagen Segments are shown in orange and indicated by the black arrowheads. The same model colored by confidence (bottom). (E) AlphaFold2 model of ERFE homotrimer bound to a BMP2/6 heterodimer colored by chain (top) and confidence (bottom). Arrows and orange color indicate Collagen Segments. (F) Predicted alignment error plots of 5 top-ranked ERFE homotrimer models (blue = small predicted error of alignment). The formation of a collagen triple helix was predicted strongly in model ranks 1 and 3 as indicated with arrows.

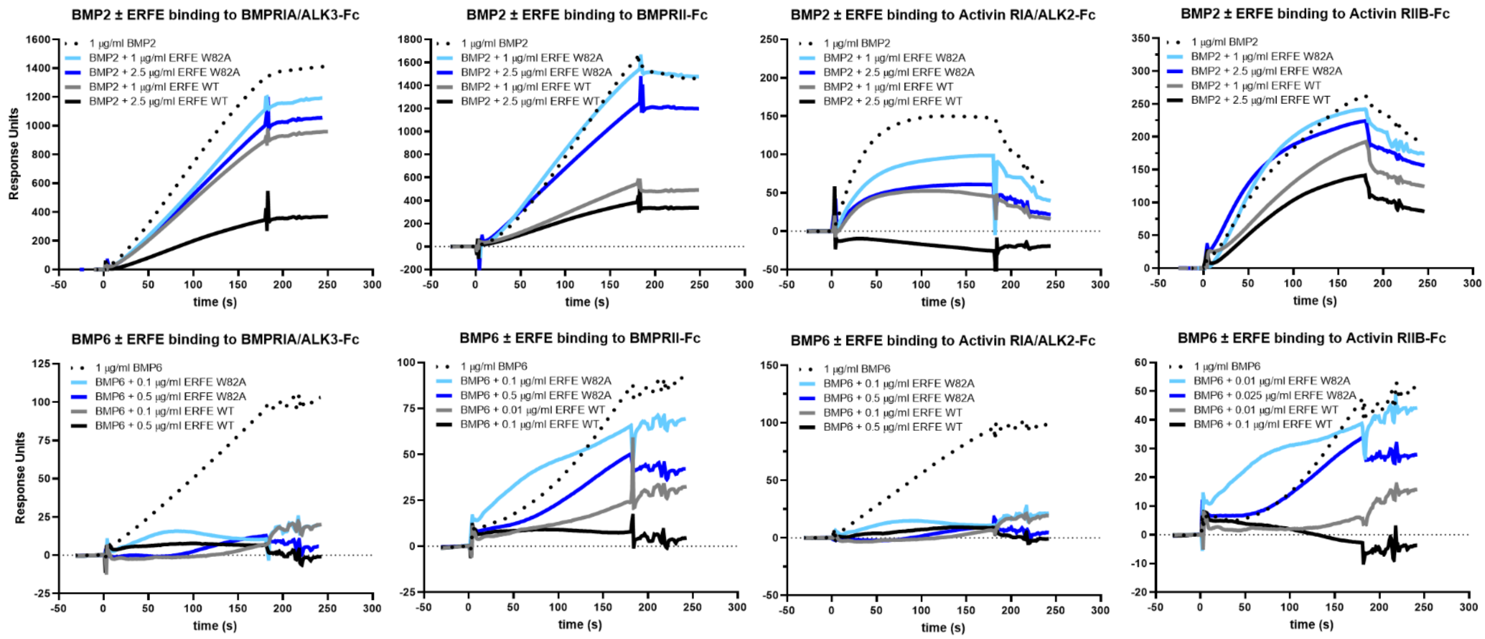

Figure S-7: Surface plasmon resonance sensorgrams of competition between ERFE and BMP receptors for binding to BMPs. Extracellular portions of different BMP receptor were immobilized and BMP2 (top) or BMP6 (bottom) flowed over alone or mixed with WT or W82A ERFE.

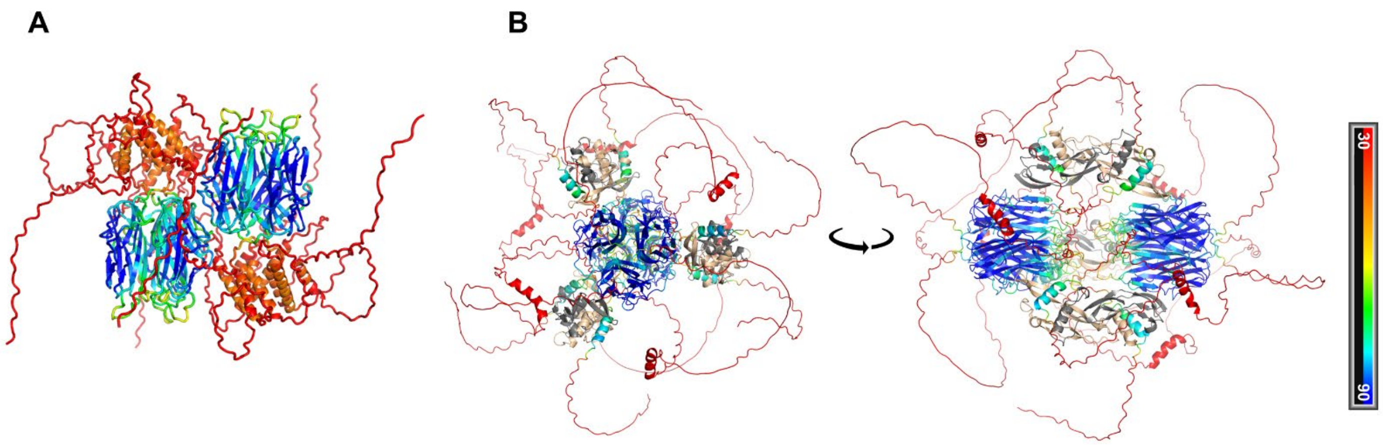

Figure S-8: (A) AlphaFold2 docking of hexameric human ERFE colored by confidence (blue = high confidence). (B) AlphaFold2 docking of hexameric human ERFE bound to three BMP2/6 dimers colored by confidence. BMP2/6 are shown in beige and grey, respectively.

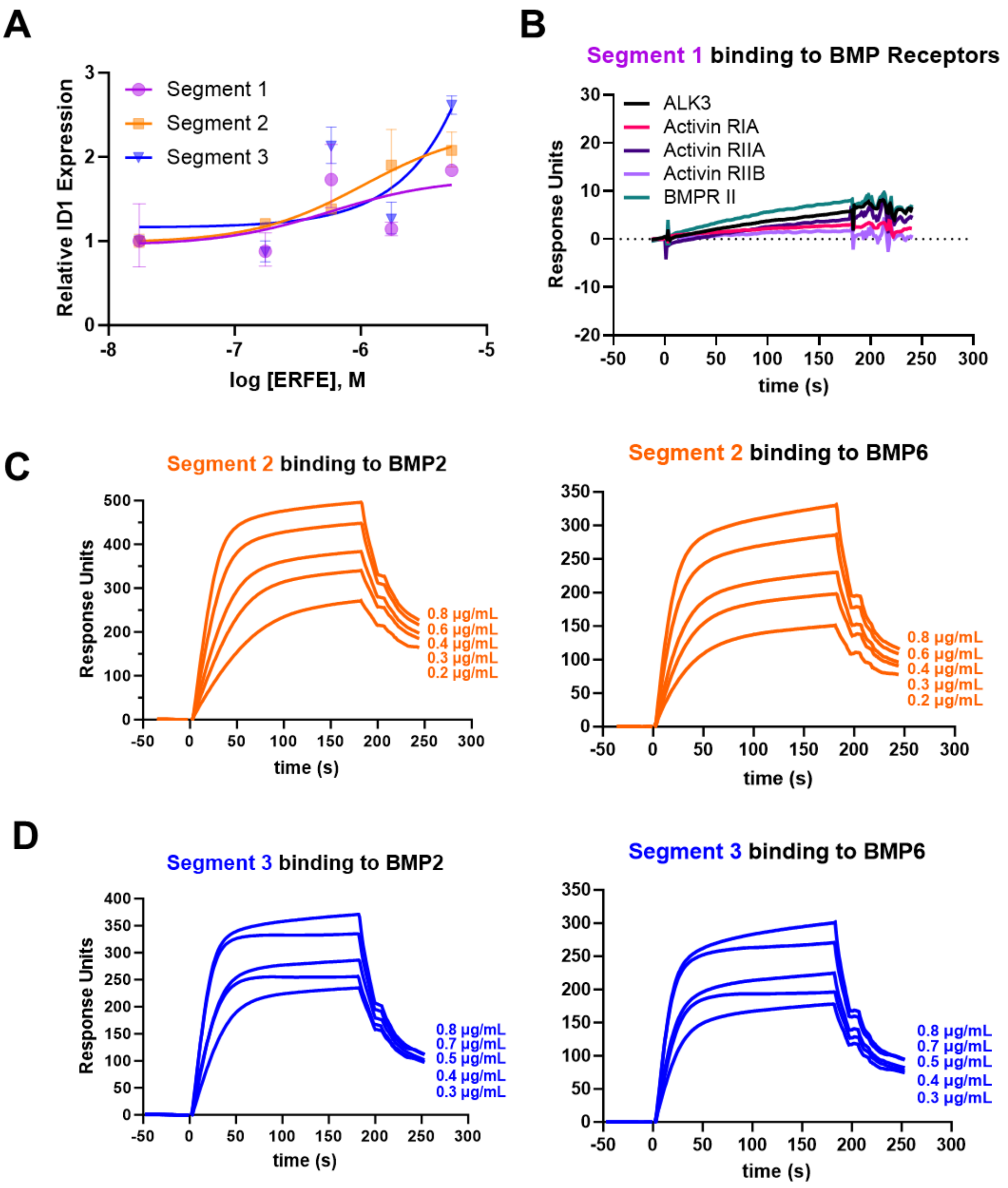

Figure S-9: (A) Expression of ID1 mRNA in Hep3B cells treated with indicated concentrations of N-terminal ERFE segments. qPCR data are normalized to untreated controls. N = 3 biological replicates. (B) Surface plasmon resonance sensorgram of N-terminal ERFE Segment 1 binding to BMP receptors. (C) SPR sensorgrams of N-terminal ERFE Segment 2 binding to BMP2 (left) and BMP6 (right). (D) SPR sensorgrams of N-terminal ERFE Segment 3 binding to BMP2 (left) and BMP6 (right).

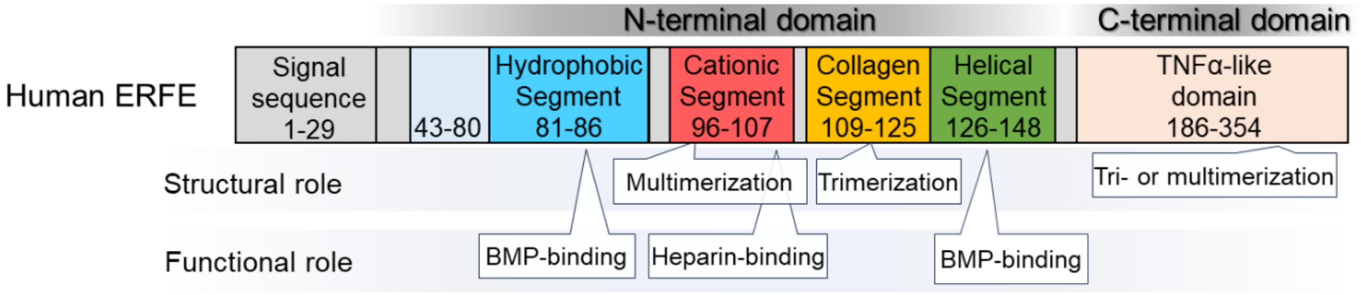

Figure S-10: Diagram of experimentally-determined roles of ERFE domains and features.
